# Designing for aging on the move: a user-needs-driven approach to an optimized recreational vehicle (RV) scheme for older Chinese using an integrated Kano–AHP–QFD framework

**DOI:** 10.3389/fpubh.2026.1781423

**Published:** 2026-04-14

**Authors:** Yin Jing, Sheng Yu, Yongsheng Cheng, Xinxin Yang

**Affiliations:** 1 School of Future Design, Beijing Normal University, Zhuhai, China; 2 School of Design & Innovation, Xiamen University Tan Kah Kee College, Zhangzhou, China; 3 School of the English Language & Culture, Xiamen University Tan Kah Kee College, Zhangzhou, China

**Keywords:** AHP, Kano model, QFD, RV design, travel-based older adult care

## Abstract

**Introduction:**

Against the backdrop of an aging population and ongoing innovations in care models for older adults, travel-based retirement—an emerging lifestyle that integrates tourism and senior living—calls for age-friendly recreational vehicle (RV) designs to meet the diverse residential and mobility needs of older adults. In such mobile retirement contexts, this study aims to produce an optimized RV design scheme that enhances living comfort and functional adaptability for older adult users.

**Methods:**

First, data were gathered through online questionnaires and field interviews to uncover the core needs of older adult RV users. Then, these needs were classified using the Kano model into five types: must-be, one-dimensional, attractive, indifferent, and reverse. Next, the Analytic Hierarchy Process (AHP) was used to prioritize these needs within a demand evaluation system. Subsequently, Quality Function Deployment (QFD) was applied to construct a House of Quality that maps user needs to product design characteristics and identifies key design indicators.

**Results:**

Based on the integrated analysis, we distilled six core design elements and developed three RV schemes tailored to older adult users. Finally, user evaluations helped determine the optimal scheme. Our findings demonstrate that the combined Kano–AHP–QFD approach effectively quantifies the demands of older adult users, enhancing design rigor and user satisfaction.

**Discussion:**

This methodological framework shows strong practical value in age-friendly RV development and offers a foundation for integrating intelligent technologies and human-centered care into future travel-based retirement schemes.

## 1 Introduction

By 2050, about one in five people worldwide, according to UN projections, will be aged 65 or older (1). China exemplifies this global trend: its over-65 population will surge from 172 million (12 percent) today to an estimated 366 million (2)—over 30% of the total population (3). This shift, further complicated by the “4 grandparents + 2 parents + 1 child” family structure resulting from the three-decade-long one-child policy (4), drives older adults toward homogenized institutional services that often neglect emotional and experiential needs (5).

Against this background, travel-based retirement models in China, especially RV-based lifestyles, show promise for countering loneliness and depression (6, 7), sharpening mental function (6, 8), and enhancing existential well-being (9). Meanwhile, China has in recent years introduced a suite of national-level policies addressing campsite approval, utility access, and road connectivity, leading to a surge in RV campsites and improved public service facilities, which in turn drives the standardization of camping tourism services (10). Consequently, the Chinese RV market grew by 24.3% in 2023, an upward trend in tandem with America (11) and Europe (12). Notably, around 60% of RV buyers at recent China-held exhibitions were seniors (13), though the current Chinese and global RV manufacturing industry remains largely oriented toward younger users (14, 15), falling short of age-friendly standards in aspects such as spatial layout and functional configuration (16). Common issues include narrow interior walkways, complex control interfaces, and safety hazards in bathroom areas—revealing a general lack of consideration for age-related physical and cognitive changes among older RV users (17). This is particularly true for those craving cross-regional traveling (18), as age-related declines in fluid intelligence (19)—especially spatial cognition and navigational abilities—can significantly challenge older adults travelers’ ability to safely and effectively navigate unfamiliar environments.

To further elucidate the scholarly advances in RV design for older adults, we review the existing literature and found it spans four main dimensions: user needs identification, spatial improvement, design method innovation, and the integration of intelligent technologies.

Regarding user needs identification, international research has increasingly emphasized the diversification of older RV user needs. Marques & Rodrigues (12), for example, categorized European motorhome travelers into “Enjoyers,” “Seekers,” and “Vacationers,” highlighting distinct preferences for travel duration, activity types, and social interaction. Tretter et al. (18) conducted a qualitative study on American RV-dwelling nomads, revealing critical unmet needs in healthcare access, including emergency medical response and chronic disease management during long-distance travel. Kim and Sivangula (20) further identified safety concerns among older drivers, such as reduced spatial cognition and navigational abilities, which directly impact RV travel confidence. In the Chinese context, Xia and Cao (21) analyzed their characteristics and travel expectations, and proposed targeted strategies for designing RVs for the “new generation of older RV users”, a term originating in Italy, though the completely narrative-based user needs restrict the possibility to operationalize quantifiable design metrics for systematic design development. Li (22) explored the software-hardware integration and multi-modal interaction design of shared RVs from a user-centered perspective, offering tactical-level, experience-driven design optimization suggestions. In a nutshell, despite their insights and inspiration for us, these studies are mainly non-empirical or non-experimental personal perspectives, thus compromising the reliability and reproducibility of the design process. A data-based study in this respect is Wu (23). Through questionnaire surveys on 20 users, Wu (23) identified problems such as noise and limited storage in current RVs and recommended promoting smart features, sustainable materials, and modular function customization. While the study provides useful insights into user preferences and design trends, its post-design focus, lack of methodological clarity, and limited engagement with recent literature constrain its academic robustness.

As regards RV spatial improvement, very limited research in the international contexts is available. In the Chinese context, Chen et al. (24) improved the ergonomic layout of kitchen spaces in a C-type RV, providing valuable guidance for future spatial design. Lin et al. (25), drawing on the Affordance Theory, analyzed cognitive, sensory, behavioral, and functional affordances, and proposed principles of comfort, usability, and contextual relevance for interior space. These space-focused studies, though, ignore user demands and lacks implications for the overall vehicle design, including interactive interface, of an age-friendly RV. Notably, Niu et al. (26), published in an international journal, presented a reductionist, space-efficiency focused approach that prioritizes quantitative optimization of movement paths over demographic-specific design considerations, offering a technically rigorous but functionally generic scheme lacking the nuanced understanding of user diversity required for inclusive senior mobility environments.

Design methods and integration of intelligent technology have also been primarily investigated in the Chinese context. In terms of design methods, Wang et al. (14), employed a comparative and practice-based CAD-driven design approach to develop a RV tailored to the emerging Chinese market. More recently, researchers have adopted innovative design methods of designing RVs. Xu et al. (27), for example, addressed design homogeneity by employing an Analytic Hierarchy Process (AHP) model, expert scoring, and fuzzy comprehensive evaluation to identify and refine optimal design schemes. Tu and Li (28) combined the Fuzzy Analytic Hierarchy Process (FAHP) with the Function-Behavior-Structure (FBS) model to analyze user needs and optimize human-machine interactive interfaces. While these studies have made notable contributions to RV design methodologies and partial RV design, they lack a closed-loop, full-chain design process that systematically identifies, prioritizes, and translates user needs into spatial planning, styling, and interactive interfaces through an integrated framework. As for integration of intelligent technology, Zhang et al. (16) incorporated augmented reality (AR) into RV personalization, enabling users to customize interiors and thereby opening new avenues for the development of the RV industry. This study, however, primarily focuses on how AR can support VR platform optimization, without addressing age-friendly, whole-vehicle customization.

Overall, current research underscores that user needs are central to product design. Nonetheless, there remains a wide gap: the design of RVs for the older adults still leaves considerable room for improvement, and most existing evaluation systems overlook detailed and systematic user experience feedback. These gaps motivated us to propose an integrated design framework. Our approach centers on the older adult’s physiological and behavioral characteristics, combining the Kano model, Analytic Hierarchy Process (AHP), and Quality Function Deployment (QFD). In doing so, we aim to address two key research questions (RQs):

*RQ1*: How can older RV users’ needs be systematically identified and prioritized to uncover key pain points in RV design?

*RQ2*: How can the needs–function mapping process be enhanced to effectively transform user demands into design schemes for older-adult-friendly RVs?

Through a structured process of “needs identification – priority quantification – function mapping,” we aim to establish a replicable demand-driven optimization pathway for RV design.

## 2 Theoretical framework and design flow

### 2.1 Kano model

**Figure 1:**
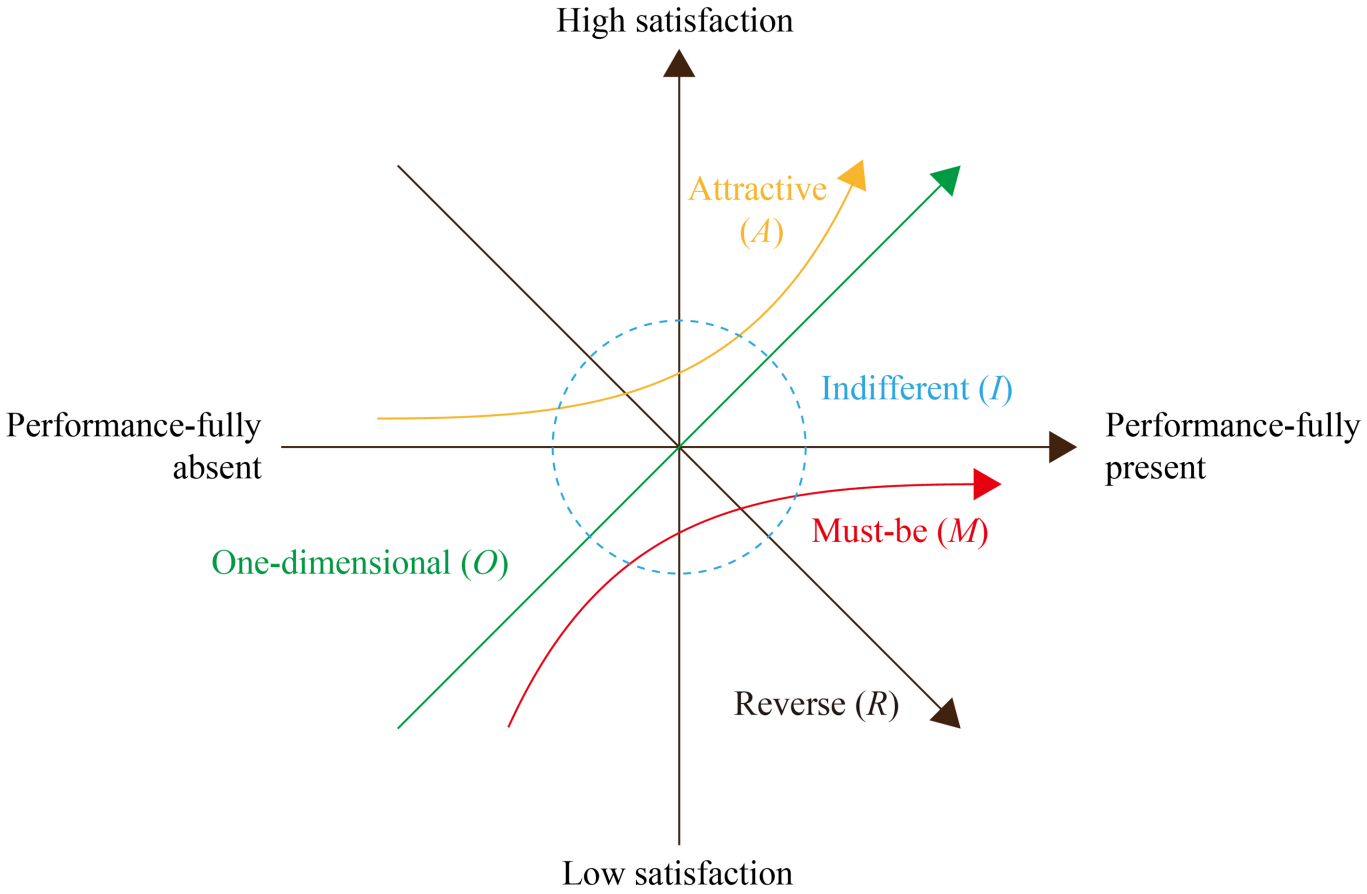
The Kano model.

The Kano model is a tool that classifies user needs into five categories of attributes—must-be (M), one-dimensional (*O*), attractive (*A*), indifferent (*I*), and reverse (*R*) (29) (Figure 1), unlike Kansei Engineering, which captures users’ affective impressions and maps them to concrete design parameters (30). The Kano model enables a systematic identification of needs priorities and reveals the relationship between product attributes and user satisfaction (31), and have therefore been used in multiple disciplines, particularly in product or service development and optimization (32). Many researchers have incorporated this model into more sophisticated models for design, such as smart product design through a Fuzzy Kano–AHP–DEMATEL–QFD approach (33), and smartphone and camera design through a Kansei-integrated Kano model (34). In the field of age-friendly design, the model has informed many strategies (35–38). Take Li et al.’s (35) research for example. Utilizing the IPA-Kano model, they quantified the needs and perceptions of the older adults and explored the key factors affecting the age-friendly design of Chengdu East Railway Station. This study, though, fails to produce actionable design schemes. More enlightening is Wang et al.’s (38) research, which designed a walker product under the combined Kano–AHP–FCE framework. A more relevant study is Jing et al.’s (39) research on designing an daily-use electric vehicle for the older adults. These existing studies on the Kano model have proven its effectiveness in accurately identifying the pain points of older RV users and supporting well-informed design decisions, thus guiding the present study on how to design an age-friendly RV.

### 2.2 AHP model

The Analytic Hierarchy Process (AHP) enables quantitative analysis for multi-criteria decision-making by constructing a hierarchical structure consisting of layers of the goal, criteria, and alternatives, supported by pairwise comparison matrices and consistency checks (40, 41). Its scientific rigor and reliability have made it widely applicable in the study of complex problems (42, 43). In the context of age-friendly design, AHP is often integrated with other techniques to enhance decision-making efficiency [e.g., (33, 38, 44, 45)]. For example, employing the AHP, along with QFD and Kansei Engineering (KE), Li and Li (44) developed an older RV-user-tailored home-use intelligent blood glucose meter that not only meets users’ physiological and psychological needs but also provides an operationally convenient, health-conscious, and esthetically pleasing experience. These practical applications demonstrate AHP’s notable strengths in balancing multidimensional demands and integrating both subjective and objective weightings, thereby providing systematic decision support for our age-friendly RV design.

### 2.3 QFD theory

Quality Function Deployment (QFD), developed by Mizuno and Akao (46), is a structured methodology that translates user needs into engineering parameters using its core tool—the House of Quality. By prioritizing user needs and mapping them through correlation matrices, QFD establishes a clear link between customer expectations and design elements during product development, thereby making products more relevant and user-centered. In the field of age-friendly design, QFD has evolved into multidimensional application frameworks, such as AHP–QFD–KE for designing age-appropriate smart blood glucose detector (44), Kano–AHP–QFD for a control interface of passenger car center (45), and KE–QFD an age-friendly restaurant platform (47). These practices have demonstrated QFD’s methodological strengths in interdisciplinary integration, precise demand translation, and iterative product optimization, thus providing a methodological reference for our research.

### 2.4 Integrated Kano-AHP-QFD framework

By integrating three complementary methods, we developed a Kano–AHP–QFD model. This model forms a complete process from user needs investigation to product design and development. We began with the Kano model to classify user needs—collected through questionnaire surveys—into five categories: must-be, one-dimensional, attractive, indifferent, and reverse. This classification helped us understand how different types of needs influence user satisfaction. However, since the Kano model does not quantify the priority of each needs (48), we incorporated the Analytic Hierarchy Process (AHP) to address this gap. By constructing pairwise comparison matrices, we quantified the relative importance of each user needs, which allowed us to prioritize them scientifically and identify key directions for design optimization.

**Figure 2:**
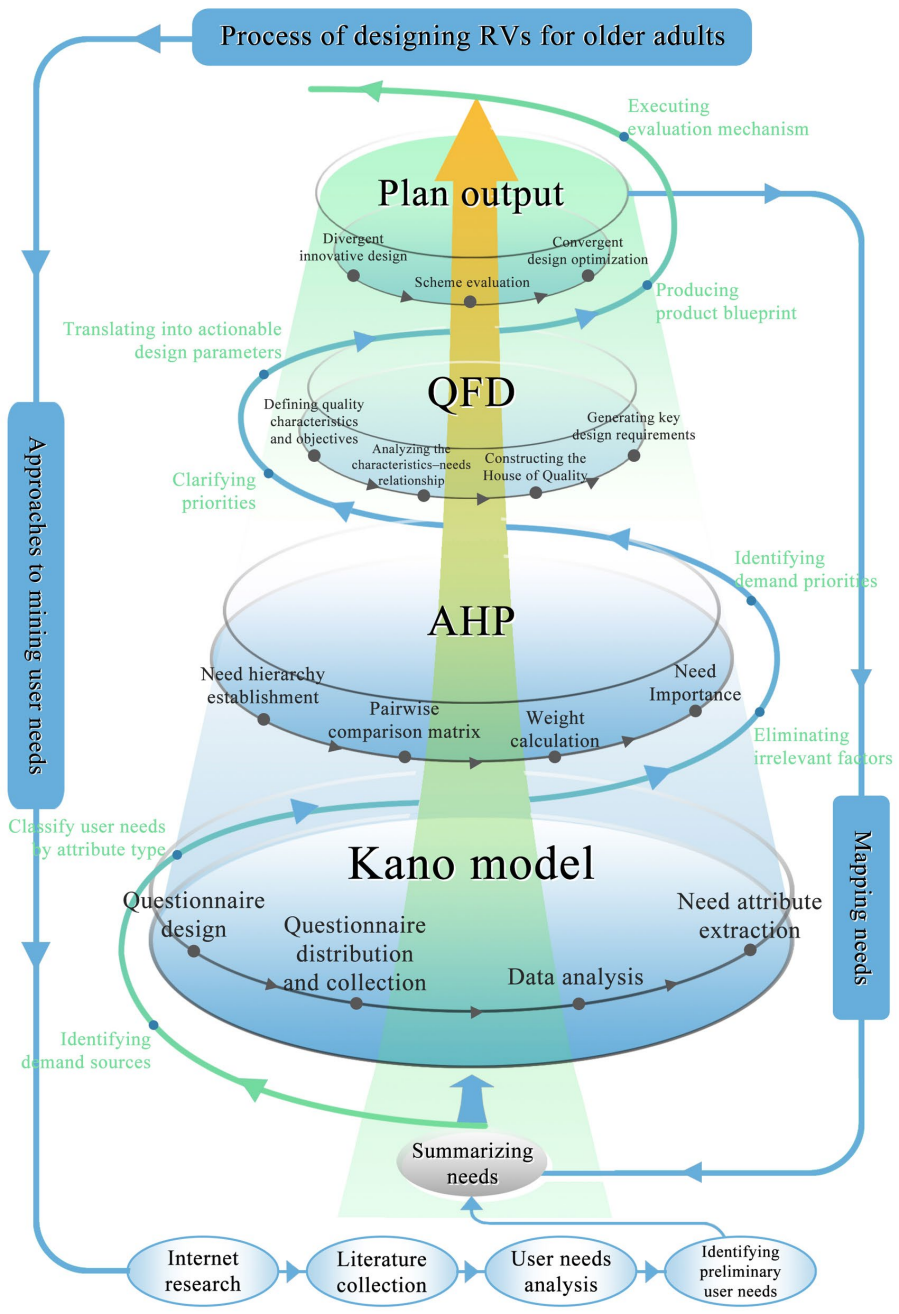
A flow chart of age-friendly RV design.

Despite the effectiveness of the Kano–AHP combination in identifying user needs and their priorities, it still lacked a way to translate these needs into concrete design parameters. To solve this problem, we applied Quality Function Deployment (QFD). We used the AHP-generated weights to build a correlation matrix that connects user expectations with specific product design features. This approach enabled us to define technical needs based on user priorities and focus the design process on what matters most to older RV users. Ultimately, our integrated model helped us transform user expectations into actionable design schemes and provided a solid foundation for targeted, user-centered product development. The overall design process is shown in Figure 2.

## 3 Research process

### 3.1 Initial needs analysis based on the Kano model

#### 3.1.1 Acquiring user needs

**Figure 3:**
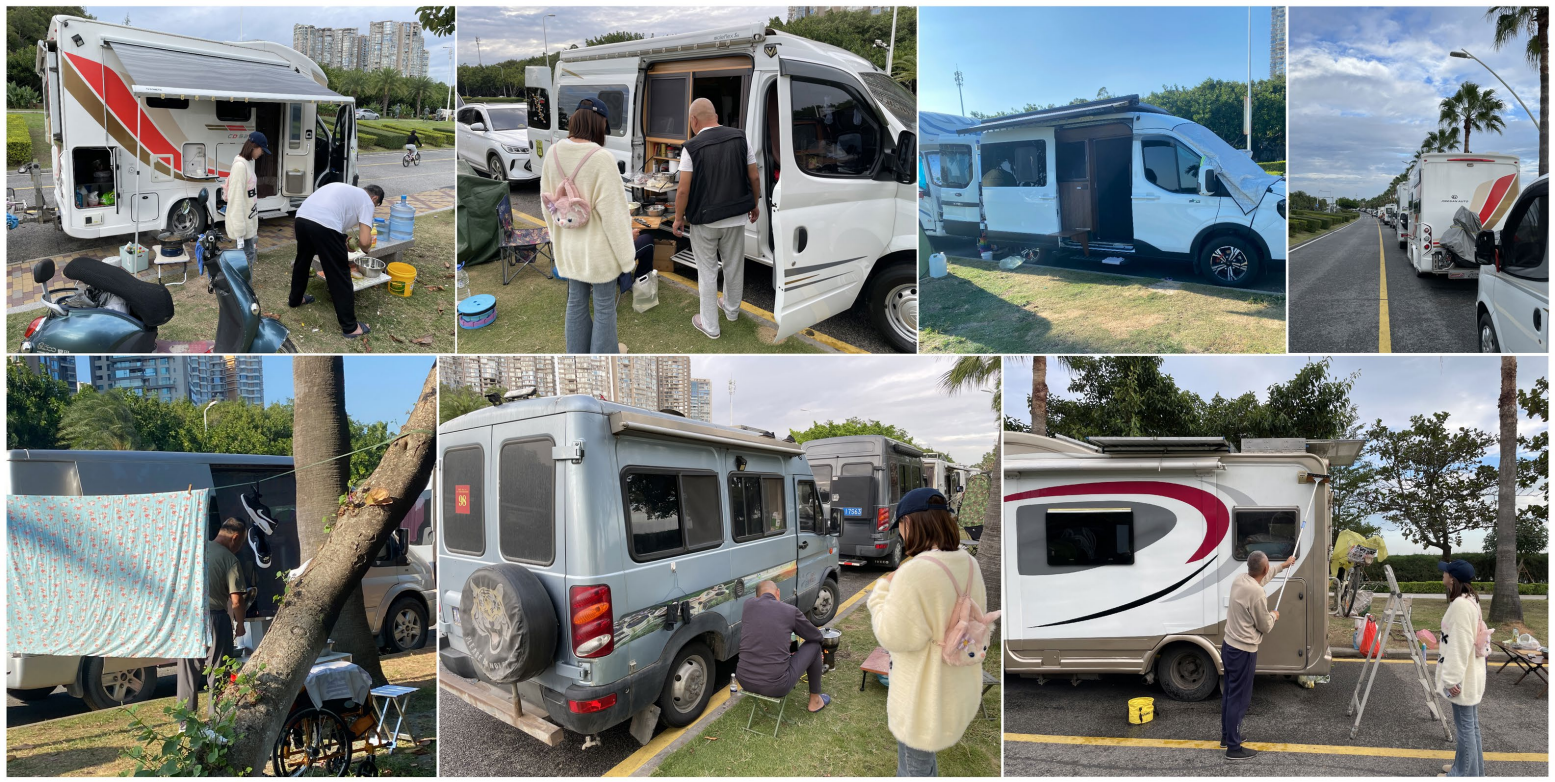
On-site interviews.

During the needs acquisition phase for older adult RV design, we focused on individuals aged 60 and above with prior RV driving experience to ensure authentic and reliable data. We first identified preliminary user needs through internet research and a review of relevant literature [e.g., (10, 13, 27, 38)]. To obtain rich, contextualized insights, we conducted, with the help of both video and audio recordings (see Figure 3), in-depth field interviews with older adults participants, most of whom had driven a long way from North China to Southeast China’s Xiamen city. We employed convenience sampling, selecting participants based on geographical proximity and their willingness to participate, which allowed us to carry out face-to-face interviews. Compared with online or remote interviews, these on-site filmed and recorded interviews provided the higher rapport and empathy (49), opportunity to directly observe participants’ interactions with RV features and to capture nuanced needs related to daily living, functional usage, and driving experience, ensuring more authentic and detailed data (see Figure 3).

To ensure the authenticity and validity of user needs data, we established clear inclusion and exclusion criteria for participant recruitment. The inclusion criteria were as follows: first, participants were aged 60 years or older; second, they had at least one experience of RV travel lasting 3 days or longer, to ensure they were fully familiar with RV functional scenarios and actual usage needs (20); third, they had clear expression ability, could independently communicate their usage experience and needs without assistance from others; fourth, they voluntarily participated in the study and provided oral informed consent. Given the minimal-risk nature of our study and potential variability in literacy and visual acuity among older adults participants, oral informed consent was adopted to reduce cognitive burden and ensure substantive comprehension. Correspondingly, the exclusion criteria included participants suffering from severe visual, hearing or motor dysfunction that prevented normal communication or operation of RV facilities, those who were unable to clearly express their personal opinions and experiences, and those with no RV travel experience or only short-term experience of less than 3 days. Consequently, we interviewed 15 participants.

The recorded materials were transcribed into text using TurboScribe,^1^ and subsequently verified manually. Key user needs were extracted using MAXQDA. To ensure the reliability of this process, we achieved a high level of inter-coder agreement (Cohen’s *κ* = 0.94). Disagreements were resolved through discussion with a third expert. Finally, we conducted member checking by returning the extracted needs to the original interviewees for confirmation and clarification (Table 1).

**Table 1:** User needs for older adult RV design.

Primary needs	Secondary needs	Explanation
*N1* Living	*n*_1_ Comfortable to sleep in	The bed area is comfortable and spacious and has privacy.
	*n*_2_ Toilet ventilation	Toilet has air exchange and ventilation.
	*n*_3_ Separate bathroom	The shower area has a separate space (wet and dry separation).
	*n*_4_ Non-slip toilet flooring	The toilet area features a non-slip design.
	*n*_5_ Separate kitchen	The kitchen area in the car has a separate enclosed space (to prevent fumes from spreading)
	*n*_6_ Spacious kitchen	The kitchen area is spacious and can be operated by two people efficiently.
	*n*_7_ Organized storage	Inside the RV, there is a well-organized storage space.
	n_8_ Spaciousness	It is easy to walk and turn around in the RV, and it is not crowded when placing wheelchairs and other assistive devices.
	*n*_9_ Air-drying	The RV has an interior area for drying clothes
	*n*_10_ Window lighting	Large windows in the RV can meet the needs of lighting and ventilation.
	*n*_11_ Mechanical ventilation	The RV is equipped with mechanical ventilation
*N2* Use	*n*_12_ Intelligent applications	In-vehicle equipment and facilities can be intelligently controlled (e.g., voice, APP control.)
	*n*_13_ Outdoor space	Outdoor space that can meet the needs of drinking tea and playing chess, fitness and exercise (e.g., awnings, outdoor tables and chairs, etc.)
	*n*_14_ Leisure and space	The RV has a leisure area with comfortable seats, convenient access and a spacious area.
	*n*_15_ Area needs	The leisure area can meet the needs of meeting guests, working, eating and other daily needs.
	*n*_16_ Two-person co-mobility	The RV has interior activity space for two persons (e.g., joint exercise).
	*n*_17_ Independent space for two	The RV has a separate interior space for two people
	*n*_18_ Medical supplies	The RV supports refrigerated storage of medicines, and has space for first aid equipment, wheelchair crutches, and health testing equipment.
	*n*_19_ Space for pets	The RV has an interior space for pets to rest and interact with pets.
*N3* Safety	*n*_20_ Emergency call	The RV is equipped with an emergency call system (can quickly contact rescue organizations or family members).
	*n*_21_ Medical inquiry	The RV provides a medical information lookup feature (e.g., nearby hospitals, navigation.)
	*n*_22_ Activity monitoring	The RV is equipped with a system to intelligently monitor activity status (e.g., fall, fainting monitoring.)
	*n*_23_ Body security	The RV is fitted with body monitoring (monitoring the surroundings of the caravan when parked).
	*n*_24_ Good privacy	The RV prevents outsiders from prying into the vehicle.
	*n*_25_ Convenient facilities	The RV has convenient boarding and alighting facilities (e.g., handrails, pedals, low step design).
*N4* Exterior and interior	*n*_26_ Appropriate styling	Exterior styling is stable and simple.
	*n*_27_ Comfortable color scheme	Calm, atmospheric colors are used.
	*n*_28_ Suitable materials	Seat and bed materials are soft and breathable, with non-slip design.
	*n*_29_ Environment-friendly decorations	The materials in the RV are environment-friendly and non-toxic.
	*n*_30_ Easy to clean	The interior is easy to clean and wear-resistant.
	*n*_31_ Interior lighting	Interior lighting is good, and there are no dead spots at night.
*N5* Driving	*n*_32_ Vehicle monitoring	The RV includes smart monitoring function (vehicle tire pressure, fault warning).
	n_33_Vehicle Operation	Driving operation is simple and easy to understand (instrument panel display is clear; control buttons are well laid out and easy to follow).
	*n*_34_ Vehicle driving	The RV provides good driving vision (e.g., through anti-glare mirrors that reduce glare from vehicles behind).

#### 3.1.2 Kano questionnaire analysis

Following the principles of the Kano model, we designed a five-point scale questionnaire based on the above 34 user needs indicators. Each item of the questionnaire was presented in both positive, neutral, and negative forms to capture respondents’ attitudes more comprehensively. The response options were structured as follows: “I like it that way” (5 points), “It must be that way” (4 points), “I am neutral” (3 points), “I can live with it that way” (2 points), and “I Dislike it that way” (1 point).

To maximize data coverage, we adopted a cross-platform, mixed-mode distribution strategy. Specifically, questionnaires were shared online via targeted communities such as the “Exchange Group for RV Self-driving Traveling” and offline in campgrounds. As a result, we collected a total of 106 responses, 90 (84.9%) of which were valid after excluding incomplete questionnaires and uniform response bias (50).

The valid responses were subsequently processed using SPSSAU’s Kano model tool. User needs attributes were categorized in accordance with Kano’s classification method (Table 2), enabling a structured understanding of user preferences and expectations. Analyses assumed independent responses and ordinal-scale data, consistent with standard Kano model practices.

**Table 2:** Kano classification for positive/negative questions.

Function/service		Negative questions				
		I dislike it that way (1 point)	I can live with it that way (2 points)	I am neutral (3 points)	It must be that way (4 points)	I like it that way (5 points)
Positive questions	I dislike it that way (1 point)	Q	R	R	R	R
	I can live with it that way (2 points)	M	I	I	I	R
	I am neutral (3 points)	M	I	I	I	R
	It must be that way (4 points)	M	I	I	I	R
	I like it that way (5 points)	O	A	A	A	Q

A, Attractive; O, One-dimensional; M, Must-be; I, Indifferent; R, Reverse; Q, Questionable.

#### 3.1.3 Better–Worse data calculation and quadrant plotting

To further quantify the impact of each indicator on user satisfaction, we calculated the Better–Worse coefficients, which evaluates how a functional requirement influences user satisfaction (51) and helps orient product development and optimization (37). Specifically, the Better value (B), also known as the satisfaction coefficient, reflects the extent to which meeting a requirement increases satisfaction. It typically ranges from 0 to 1, with values closer to 1 indicating a stronger positive impact. Conversely, the Worse value (W), or the dissatisfaction coefficient, measures the degree to which not meeting a requirement decreases satisfaction. This value usually ranges from 0 to −1, with scores nearer to −1 indicating a greater negative impact.

Using SPSSAU we calculated these coefficients based on the response distributions across the five Kano attributes. The calculation formulas are shown in Equations 1, 2, and the results of the Kano analysis for the preliminary user needs are presented in Table 3.


$$B=\frac{A+O}{A+O+M+I}$$ (1)

$$W=(-1)\times \frac{O+M}{A+O+M+I}$$ (2)

**Table 3:** Kano attributes and Better-Worse coefficients of RV user needs.

User needs indicators	*M*/%	*O*/%	*A*/%	*I*/%	*R*/%	Q/%	Attributes	B/%	W/%
*n*_1_	36.67	18.89	26.67	17.78	0.00	0.00	*M*	45.56	−55.56
*n*_2_	30.00	21.11	23.33	25.56	0.00	0.00		44.44	−51.11
*n*_10_	27.78	26.67	16.67	27.78	1.11	0.00		43.82	−55.06
*n*_13_	30.00	17.78	24.44	26.67	1.11	0.00		42.70	−48.31
*n*_15_	32.22	14.44	30.00	22.22	1.11	0.00		44.94	−47.19
*n*_32_	33.33	24.44	17.78	23.33	1.11	0.00		42.70	−58.43
*n*_33_	30.00	23.33	23.33	22.22	1.11	0.00		47.19	−53.93
*n*_34_	27.78	18.89	26.67	25.56	1.11	0.00		46.07	−47.19
*n*_3_	16.67	35.56	27.78	20.00	0.00	0.00	*O*	63.33	−52.22
*n*_7_	20.00	40.00	21.11	18.89	0.00	0.00		61.11	−60.00
*n*_14_	20.00	35.56	24.44	20.00	0.00	0.00		60.00	−55.56
*n*_23_	14.44	41.11	27.78	15.56	1.11	0.00		69.66	−56.18
*n*_24_	6.67	50.00	21.11	21.11	1.11	0.00		71.91	−57.30
*n*_27_	7.78	36.67	24.44	31.11	0.00	0.00		61.11	−44.44
*n*_28_	14.44	42.22	18.89	24.44	0.00	0.00		61.11	−56.67
*n*_30_	15.56	35.56	26.67	21.11	1.11	0.00		62.92	−51.69
*n*_31_	11.11	42.22	21.11	24.44	1.11	0.00		64.04	−53.93
*n*_4_	14.44	21.11	46.67	16.67	1.11	0.00	*A*	68.54	−35.96
*n*_5_	17.78	17.78	43.33	21.11	0.00	0.00		61.11	−35.56
*n*_8_	11.11	20.00	46.67	21.11	1.11	0.00		67.42	−31.46
*n*_12_	10.00	13.33	48.89	27.78	0.00	0.00		62.22	−23.33
*n*_18_	7.78	22.22	53.33	16.67	0.00	0.00		75.56	−30.00
*n*_20_	14.44	33.33	36.67	15.56	0.00	0.00		70.00	−47.78
*n*_21_	12.22	24.44	44.44	17.78	1.11	0.00		69.66	−37.08
*n*_25_	10.00	24.44	46.67	18.89	0.00	0.00		71.11	−34.44
*n*_26_	6.67	25.56	40.00	26.67	1.11	0.00		66.29	−32.58
*n*_6_	12.22	16.67	27.78	42.22	1.11	0.00	*I*	44.94	−29.21
*n*_11_	15.56	18.89	23.33	42.22	0.00	0.00		42.22	−34.44
*n*_16_	13.33	21.11	24.44	40.00	1.11	0.00		46.07	−34.83
*n*_17_	15.56	17.78	25.56	40.00	1.11	0.00		43.82	−33.71
*n*_19_	13.33	15.56	21.11	45.56	4.44	0.00		38.37	−30.23
*n*_22_	13.33	24.44	23.33	37.78	1.11	0.00		48.31	−38.20
*n*_9_	14.44	42.22	18.89	24.44	0.00	0.00		61.11	−56.67
*n*_29_	12.22	33.33	12.22	41.11	1.11	0.00		46.07	−46.07

According to the Kano attributes of 34 user needs in Table 3, there were 8 *M*, 9 *O*, 9 *A*, and 8 *I* attributes.

*M* included *n*_1_ (comfortable to sleep in), *n*_2_ (toilet ventilation), *n*_10_ (window lighting), *n*_13_ (outdoor space), *n*_15_ (area needs), *n*_32_ (vehicle monitoring), *n*_33_ (vehicle operation) and *n*_34_ (vehicle driving). When the degree of perfection of these needs was high, user satisfaction rose only slightly; otherwise, it decreased significantly. The 8 *M* attributes are consistent with the “safety first, practicality-oriented” demand logic shaped by age-related physiological decline. This explains the core reason why existing RV designs oriented toward younger users, which often prioritize entertainment features and stylistic differentiation, fail to meet the needs of older populations.

*O* included *n*_3_ (separate bathroom), *n*_7_ (organized storage), *n*_14_ (leisure and space), *n*_23_ (body security), *n*_24_ (good privacy), *n*_27_ (comfortable color scheme), *n*_28_ (suitable materials), *n*_30_ (easy to clean), and *n*_31_ (interior lighting). When these needs were perfected to a high degree, user satisfaction increased, and vice versa, it decreased.

*A* included *n*4 (non-slip toilet flooring), *n*_5_ (separate kitchen), *n*_8_ (spaciousness), *n*_12_ (intelligent applications), n_18_ (medical supplies), *n*_20_ (emergency call), n_21_ (medical inquiry), *n*_25_ (convenient facilities) and *n*_26_ (appropriate styling). When these needs were well developed, user satisfaction increased significantly, and vice versa, the decrease was insignificant.

*I* included *n*_6_ (spacious kitchen), *n*_11_ (mechanical ventilation), *n*_16_ (two-person co-mobility), *n*_17_ (independent space for two), *n*_19_ (space for pets), *n*_22_ (activity monitoring), *n*_9_ (air-drying), and *n*_29_ (environment-friendly decorations). There was no significant relationship between these needs and satisfaction. The quadrant analysis graph of Better–Worse coefficient can visualize the distribution of needs attributes for detailed analysis based on the comparison of 34 needs indicators.

### 3.2 User needs weight analysis based on AHP

#### 3.2.1 Establishing a hierarchical model

While the Kano model is effective in categorizing user demands for age-friendly RV design, it does not clearly prioritize these needs in terms of importance. To identify key areas of focus in the design process, we integrated the Kano model with the Analytic Hierarchy Process (AHP), a widely used method in product development. By consulting five experts in the filed of design, we assigned weights to each need, enabling a structured calculation and ranking of user needs.

**Figure 4:**
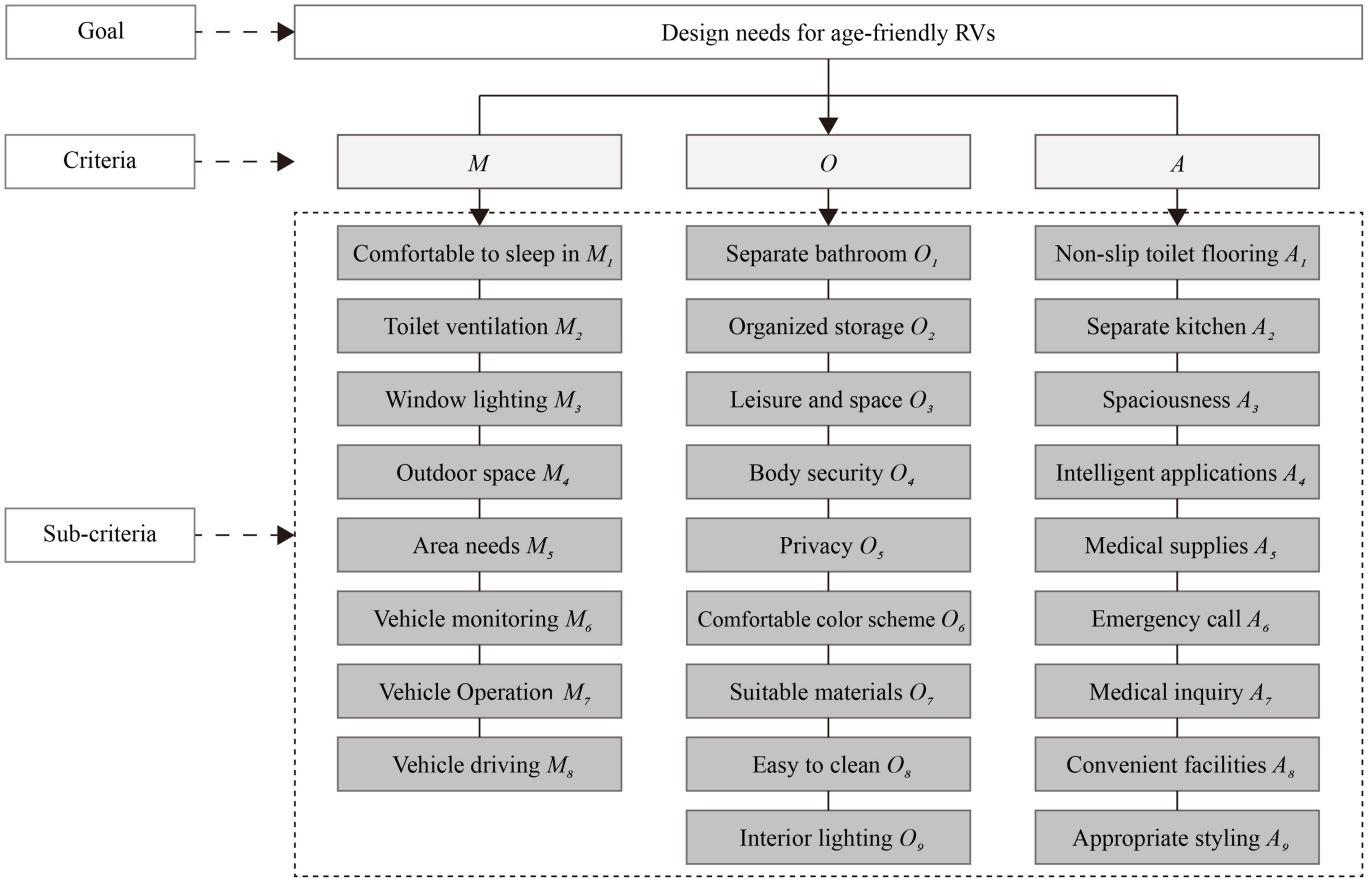
A hierarchical structure model of design needs for age-friendly RVs.

Notably, among the above-mentioned four Kano-based groups of user needs, we excluded the *I* attributes from the current analysis as they exhibited no clear relationship with user satisfaction. As a result, only those classified under *M*, *O*, and *A* attributes were used to construct the hierarchical framework for age-friendly RV design (Figure 4). In Figure 4, level 1 (goal) of the hierarchy defines the core objective and decision-making orientation, which, in this study, is the design of older-adult-friendly RVs. Level 2 (criteria) identifies key design principles necessary to achieve this objective, which, in this study, are represented by the *M*, *O*, and *A* attributes. Level 3 (sub-criteria) specifies the concrete design elements and actionable strategies.

#### 3.2.2 Establishing judgment matrices

To establish the weights of each design element, we invited an panel of 20 evaluators, including 15 older adults with RV driving experience, 2 automotive designers, and 3 professors of design. Via pairwise comparison-based scoring on a scale of 1–9, we constructed a judgment matrix, whose scale and explanation are detailed in Table 4.

**Table 4:** Scale and explanation of the judgment matrix.

Scale	Explanation
1	Indicator a is as important as indicator b
3	Indicator a is slightly more important than indicator b
5	Indicator a is significantly more important than indicator b
7	Indicator a is more strongly important than indicator b
9	Indicator a is extremely more important than indicator b
2,4,6,8	Intermediate values of the above neighboring judgments
1/2,1/3,.,1/9	If indicator i is more important than indicator j on a scale of n, the opposite is 1/n.

It should be noted that to ensure the objectivity and representativeness of the evaluation results, we adopted an equal-weight aggregation strategy to integrate evaluators’ pairwise comparison matrices. This approach was chosen to balance the perspectives of different stakeholder groups (older RV users, designers, and professors) and ensure that no single group dominated the decision-making process (52). Additionally, given the differing backgrounds and roles of the evaluators, we expected heterogeneity in preference structures that potentially lead to variability in pairwise comparison judgments. For instance, older RV users may prioritize safety and comfort, designers may emphasize spatial configuration and esthetics, while professors may focus on technical feasibility and methodological robustness. Such inter-evaluator variability, inherent in multi-stakeholder decision-making contexts, was accommodated through the structured aggregation mechanism of the AHP framework.

The judgment matrix for age-friendly RV design is *X* (Equation 3).


$$X=[\begin{array}{ccc} 1.000 & 0.411 & 0.250\\ 2.433 & 1.000 & 0.244\\ 4.000 & 4.100 & 1.000 \end{array}]$$ (3)

The judgment matrices for M, O, and A attributes are shown in Equations 4–6, respectively.


$$X=[\begin{array}{ccc} 1.000 & 0.411 & 0.250\\ 2.433 & 1.000 & 0.244\\ 4.000 & 4.100 & 1.000 \end{array}]$$ (4)

$$M=[\begin{array}{cccccccc} 1.000 & 0.769 & 1.189 & 1.393 & 2.121 & 1.218 & 1.136 & 0.662\\ 1.300 & 1.000 & 1.060 & 2.063 & 2.121 & 1.280 & 1.174 & 0.609\\ 0.841 & 0.944 & 1.000 & 0.761 & 1.230 & 0.652 & 0.946 & 0.385\\ 0.718 & 0.485 & 1.313 & 1.000 & 0.901 & 0.407 & 0.475 & 0.261\\ 0.471 & 0.471 & 0.813 & 1.110 & 1.000 & 0.323 & 0.346 & 0.222\\ 0.821 & 0.781 & 1.533 & 2.457 & 3.100 & 1.000 & 0.502 & 0.286\\ 0.880 & 0.851 & 1.057 & 2.107 & 2.893 & 1.993 & 1.000 & 0.263\\ 1.510 & 1.642 & 2.600 & 3.833 & 4.500 & 3.500 & 3.800 & 1.000 \end{array}]$$ (5)

$$O=[\begin{array}{ccccccccc} 1.000 & 0.949 & 1.402 & 2.711 & 0.316 & 0.400 & 0.937 & 0.820 & 0.547\\ 1.053 & 1.000 & 1.394 & 2.546 & 0.375 & 0.449 & 1.376 & 0.670 & 0.811\\ 0.713 & 0.717 & 1.000 & 1.312 & 0.283 & 0.327 & 0.484 & 0.396 & 0.621\\ 0.369 & 0.393 & 0.762 & 1.000 & 0.204 & 0.327 & 0.417 & 0.382 & 0.298\\ 3.167 & 2.667 & 3.533 & 4.900 & 1.000 & 0.588 & 1.460 & 0.940 & 1.082\\ 2.500 & 2.227 & 3.060 & 3.060 & 1.700 & 1.000 & 1.448 & 1.934 & 1.369\\ 1.067 & 0.727 & 2.067 & 2.400 & 0.685 & 0.690 & 1.000 & 0.493 & 0.558\\ 1.220 & 1.493 & 2.527 & 2.617 & 1.064 & 0.517 & 2.027 & 1.000 & 0.370\\ 1.827 & 1.233 & 1.610 & 3.357 & 0.924 & 0.730 & 1.793 & 2.700 & 1.000 \end{array}]$$ (6)

#### 3.2.3 Weight calculation

When calculating the weights, we transformed subjective judgments into numerical values by comparing the importance of any two factors. The specific steps were: (1) listing the pairwise comparison results of all factors, (2) calculating the weight of each factor mathematically, and (3) verifying the rationality of the results. This method enabled us to assign weights objectively. According to the judgment matrix, we applied the geometric mean method to find out the average value *V_i_* (Equation 7).


$$V_{i}=\sqrt[{n}]{\prod_{i}^{n}s_{\mathrm{ij}}}$$ (7)

Where: *S_ij_* means *the i*th-row and *j*th-column indicators in the matrix; *n* denotes the number of indicators.

To ensure methodological consistency across all stages of analysis, we applied the same geometric mean aggregation approach uniformly to all judgment matrices (*X*, *M*, *O*, and *A*), thus ensuring the reliability and comparability of evaluation results across different attribute groups.

The results were then combined into the matrix form (Equation 8).


$$V_{z}={\left(V_{1},V_{2},V_{3},\cdots ,V_{n}\right)}^{T}$$ (8)

Next, the eigenvectors of the *X*, *M*, *O* and *A* matrices were calculated by the above formula (Equations 9–12).


$$V_{x}={\left(1.951,0.672,0.376\right)}^{T}$$ (9)

$$\begin{array}{l} V_{M}=(2.209,1.015,0.934,0.467,\\ \;\;\;\;\;\;\;\;\;\;\;\;\;\;0.568,0.731,1.089,0.987){}^{T} \end{array}$$ (10)

$$\begin{array}{l} V_{O}=(1.372,1.094,0.836,1.697,1.554,0.369,\\ \;\;\;\;\;\;\;\;\;\;\;\;\;\;0.519,0.822,0.738){}^{T} \end{array}$$ (11)

$$\begin{array}{l} V_{A}=(1.617,0.848,0.838,0.9341.299,\\ \;\;\;\;\;\;\;\;\;\;\;\;1.589,0.961,0.639,0.275){}^{T} \end{array}$$ (12)

After normalizing the calculation results, we obtained the weights of the primary indicator, denoted as *W = {w_1_, w_(2)_,.,w_n_}*. For each primary indicator, the weights of its sub-criteria were also normalized to form a sub-weight vector *W_n_ = {w_(1) (1)_, w1_(2)_,.,w_ij_}*, where *j = {1, 2,3.,n}*, thus the resulting weight vectors *W_i_* (Equation 13).


$$W_{i}=\frac{V_{i}}{\sum_{i=1}^{n}V_{i}}$$ (13)

Additionally, by normalizing the weights of each criterion layer and sub-criterion layer, we obtained the normalized weight vectors of each indicator *X*, *M*, *O*, and *A* (Equations 14–17).


$$W_{x}={\left(0.650,0.224,0.125\right)}^{T}$$ (14)

$$\begin{array}{l} W_{M}=(0.276,0.127,0.117,0.058,\\ \;\;\;\;\;\;\;\;\;\;\;\;\;\;\;0.071,0.091,0.136,0.123){}^{T} \end{array}$$ (15)

$$\begin{array}{l} W_{O}=(0.152,0.122,0.093,0.189,0.173,\\ \;\;\;\;\;\;\;\;\;\;\;\;\;0.040,0.058,0.091,0.082){}^{T} \end{array}$$ (16)

$$\begin{array}{l} W_{A}=(0.180,0.094,0.093,0.103,0.144,\\ \;\;\;\;\;\;\;\;\;\;\;\;\;\;0.177,0.107,0.071,0.030){}^{T} \end{array}$$ (17)

Through these calculations, we obtained the comprehensive weight values of each user needs indicator, namely: comfortable to sleep in > vehicle operation > toilet ventilation > vehicle driving > window lighting > vehicle monitoring > area needs > body security > privacy > outdoor space > separate bathroom > organized storage > non-slip toilet flooring > emergency call > leisure and space > easy to clean > interior lighting > medical supplies > medical inquiry > intelligence > suitable materials > separate kitchen > spaciousness > comfortable color scheme > convenient facilities > appropriate styling, as shown in Table 5.

**Table 5:** AHP-based weight values of user needs indicators for age-friendly RVs.

Criteria level	Weights of the criteria level	Sub-criteria level	Weights of the sub-criteria level	Combined weights	Ranking of weights
*M*	0.650	*M*_1_	0.276	0.179	1
		*M*_2_	0.127	0.083	3
		*M*_3_	0.117	0.076	5
		*M*_4_	0.058	0.038	10
		*M*_5_	0.071	0.046	7
		*M*_6_	0.091	0.059	6
		*M*_7_	0.136	0.088	2
		*M*_8_	0.123	0.080	4
*O*	0.224	*O*_1_	0.152	0.034	11
		*O*_2_	0.122	0.027	12
		*O*_3_	0.093	0.021	15
		*O*_4_	0.189	0.042	8
		*O*_5_	0.173	0.039	9
		*O*_6_	0.041	0.009	24
		*O*_7_	0.058	0.013	21
		*O*_8_	0.091	0.020	16
		*O*_9_	0.082	0.018	17
*A*	0.125	*A*_1_	0.180	0.023	13
		*A*_2_	0.094	0.012	22
		*A*_3_	0.093	0.012	23
		*A*_4_	0.104	0.013	20
		*A*_5_	0.144	0.018	18
		*A*_6_	0.177	0.022	14
		*A*_7_	0.107	0.013	19
		*A*_8_	0.071	0.009	25
		*A*_9_	0.031	0.003	26

As can be seen from Table 5, the weight distribution of the criterion layer further validates the priority logic of older-adult-friendly RV design: *M* attributes account for 65.0% of the total weight, far exceeding *O* (22.4%) and *A* (12.5%) attributes. This distribution is starkly different from that of general consumer products, highlighting the uniqueness of age-friendly design, where basic functional completeness takes precedence over value-added function optimization.

The top-ranked needs indicators “comfortable to sleep in” (0.179) and “vehicle operation” (0.088) directly respond to the core pain points of existing RV designs, which generally have small bed sizes and complex central control interfaces, matching the physiological characteristics of older adults people with higher sleep quality needs and lower acceptance of complex operations. Weights of living and driving demands are significantly higher than those of appearance and interior decoration demands, indicating that older RV users position RVs as “mobile living spaces” rather than “leisure and entertainment products”, channeling us to prioritize resource allocation to functional optimization rather than differentiated competition in appearance modeling.

#### 3.2.4 Maximum eigenroot calculation

To verify the logical rationality of the judgment matrices, we calculated the maximum eigenroot. After establishing the judgment matrices, we derived their maximum eigenvalues through mathematical operations, and then used these values to invert the weight proportion of each factor. Meanwhile, the consistency index (e.g., CR value) was calculated to rule out self-contradiction in the expert scoring. If the results meet the needs, it means scientific and credible weight allocation. The operation formula is shown in Equation 18.


$$\lambda_{\max}=\sum_{i=1}^{n}\frac{{\left(\mathrm{MW}\right)}_{i}}{nW_{i}}$$ (18)

Where *n* denotes the order of the judgment matrix; *M* means judgment matrix; and *W* is eigenvectors of judgment matrix *A*.

#### 3.2.5 Result consistency test

To ensure the logical consistency of the judgment matrices, we performed a consistency check on the calculated weights (Equations 19, 20). If the result fails the check, the ranking of criteria at each level must be re-evaluated.


$$C_{I}=\frac{\lambda_{\max}-n}{n-1}$$ (19)

$$C_{R}=\frac{C_{I}}{R_{I}}$$ (20)

Where *n* denotes the value corresponding to the evaluation scale of the judgment matrices; *C_R_* stands for consistency ratio; and *R_I_* represents consistency indicator.

When *C_R_* ≥ 0.1, it means inconsistency in the judgment matrix, and vice versa. The consistency test results of the comparison matrix of each level are shown in Table 6.

**Table 6:** Consistency test result.

Indicator	*λ*_max_	*C*_I_	*R*_I_	*C*_R_	Consistency
*X*	3.095	0.047	0.520	0.091	Passed
*M*	8.297	0.042	1.410	0.030	Passed
*O*	9.303	0.038	1.460	0.026	Passed
*A*	9.707	0.088	1.460	0.061	Passed

### 3.3 Initial needs analysis based on QFD quality house

**Figure 5:**
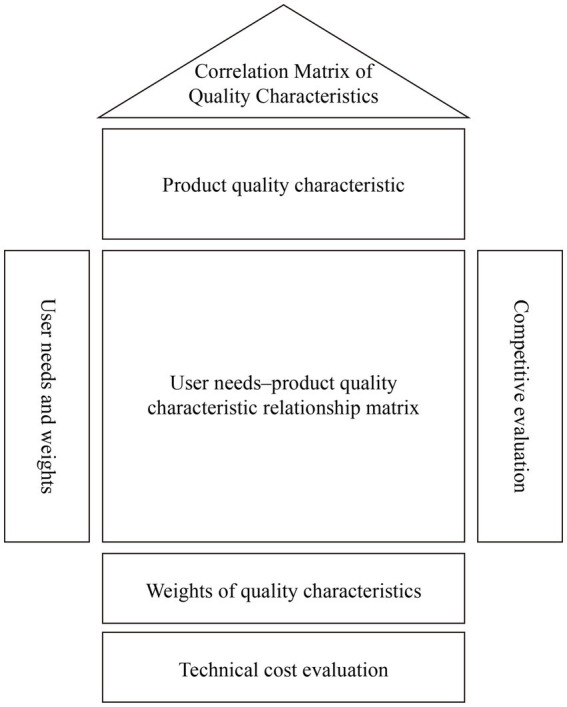
QFD quality house.

First, based on user needs and their explanations, similar items were categorized and merged. Then, these needs were matched with appropriate design features, resulting in ten key design features. The relative importance of product quality characteristics was ranked using the QFD quality house. In this method, the quality characteristics are represented as the ceiling of the house, the user needs and their weights as the walls, the correlations between user needs and quality characteristics as the interior, and the correlation weights and absolute weights of the quality characteristics as the basement (Figure 5).

A panel of experts consisting of five professors of industrial design (three specializing in transportation design and two in service design) evaluated the correlation between the user needs and quality characteristics of age-friendly RVs. Via pairwise comparison-based scoring, where a score of 5, 3, 1, and 0 indicates strong, medium, weak, and no correlation, respectively, we obtained the corresponding scores. Well-established in QFD practices (53, 66), this scoring scheme was chosen for its simplicity and clarity, providing a structured framework for experts to express the strength of relationships while minimizing ambiguity.

Then, using Equations 21, 22, we calculated the absolute and relative importance weights of the product quality characteristics (Table 7).


$$O_{j}=\sum_{i=1}^{q}O_{i}P_{\mathrm{ij}}$$ (21)

$$O_{k}=\frac{O_{j}}{\sum_{i=1}^{q}U_{j}}$$ (22)

**Table 7:** QFD correlation matrix between user needs and quality characteristics.

User needs	Quality characteristics										
	Overall weight of user needs	*d*_1_	*d*_2_	*d*_3_	*d*_4_	*d*_5_	*d*_6_	*d*_7_	*d*_8_	*d*_9_	*d*_10_
*n*_1_	0.179	5	3	1	3	1	1	1	1	1	1
*n*_2_	0.083	3	3	1	1	1	3	1		1	1
*n*_3_	0.034	3	3		1	1	3	1			1
*n*_4_	0.012	3	5		3	1	3	1	1	1	1
*n*_6_	0.027	3	5		3	1	3	1		1	1
*n*_7_	0.012	5	5		3	1	1	1		1	1
*n*_9_	0.076	3	1	1	1	1	1	1	1	1	1
*n*_10_	0.038	1	1		1	1		1	1		1
*n*_11_	0.021	3	3		1		1	1			1
*n*_12_	0.046	3	3		1	1				1	1
*n*_13_	0.013	1	1	3	3	1	1		1		1
*n*_15_	0.023	1	1		3	1				3	1
*n*_18_	0.018	1	1		3	5				1	1
*n*_20_	0.022	1	1		3	5					1
*n*_21_	0.013	1	1		3	3				1	1
*n*_23_	0.039	3	3		1		1	1	1	1	1
*n*_24_	0.009	3	3	1	1	1	1	1		1	1
*n*_25_	0.02	3	3		1		3		1	1	
*n*_26_	0.018	3	1	1	3	1	1	1	1		1
*n*_27_	0.042	3	1	1	1	1		1		3	3
*n*_28_	0.003	1	1		1		1	5	3	1	
*n*_29_	0.009	3	1		1	1	1	3	5		
*n*_30_	0.013	3	1		1	1	3	3	3	3	1
*n*_32_	0.059	1		1	3	3					5
*n*_33_	0.088			3	1	1					3
*n*_34_	0.08			5	1	1					1
Absolute importance weights of quality characteristics		2.491	1.836	1.169	1.789	1.218	0.946	0.671	0.488	0.771	1.461
Relative Importance weights of quality characteristics / %		1	3	6	2	5	7	9	10	8	4

d_1_, living comfort; d_2_, reasonable functional layout; d_3_, intelligent vehicle control; d_4_, age-friendly design; d_5_, medical & safety assurance; d_6_, easy-to-clean interior decorations; d_7_, attractive appearance; d_8_, well-coordinated colors; d_9_, suitable material structure; d_10_, vehicle safety assurance.

Where *O_j_* represents the absolute importance weight of quality characteristics, *O_i_* stands for the comprehensive weight of user demand, *P_ij_* denotes the correlation coefficient between user demand and quality characteristics, and *O_k_* means the relative importance weight of quality characteristics. The results of the weight calculation were then ranked.

### 3.4 Summary of design points

**Figure 6:**
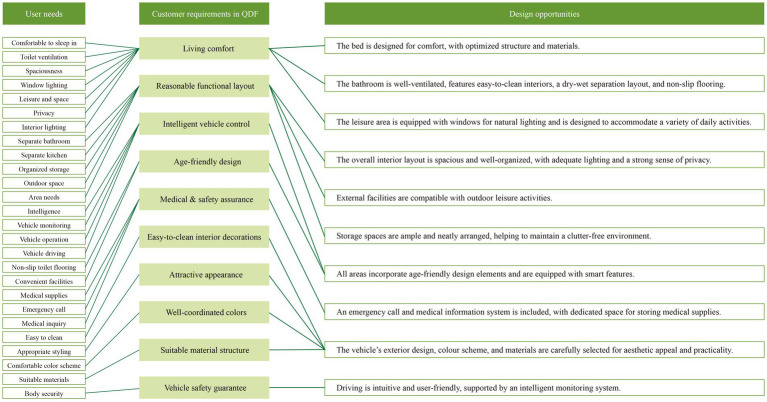
Design opportunity points.

According to the above data, we summarized the user needs and product quality characteristics and concluded the design opportunity points (Figure 6).

## 4 Design of age-friendly RVs based on Kano–AHP–QFD

### 4.1 Scheme and evaluation

#### 4.1.1 Scheme construction

The above Kano-based needs analysis identified must-be (*M*), one-dimensional (*O*), and attractive (*A*) attributes as key to enhancing user satisfaction. Accordingly, we focused on prioritizing these three types of attributes using AHP and translating the needs into product quality characteristics through QFD. Drawing on the data in Tables 5, 7, we prioritized the top-ranked design elements in this RV design.

##### 4.1.1.1 Internal space optimization

Older RV users place high demands on comfort and convenience within the living area. We therefore adopted a barrier-free design to ensure that interior passages and turns comply with accessibility standards, facilitating the movement and transfer of wheelchairs or walkers. In addition, users have specific needs for natural lighting and ventilation. Thus, we incorporated large windows and skylights to introduce sufficient daylight and create a bright, pleasant living environment. Storage space was another key concern, and the layout should be systematically and rationally planned. We therefore set appropriately placed storage cabinets and concealed compartments to provide a spacious, comfortable, and user-friendly interior.

##### 4.1.1.2 Advanced driver assistance system (ADAS)

Older adults drivers may face uncertainties such as decreased reaction speed and unstable operation during the driving process. Thus, we might consider installing advanced driver assistance system (ADAS), which has proved feasible for improve road safety for seniors (54). The adaptive cruise control, for example, was planned to automatically adjust speed based on the vehicle ahead, maintain a safe following distance, and reduce driver fatigue during long-distance travel (55). Also, an intelligent parking assistance system could be installed to reduce the stress of parking by automatically detecting parking spaces and performing assisted or fully automated parking (56, 57). Moreover, a fatigue driving monitoring system, when in place, could detect signs of fatigue by monitoring the driver’s facial expressions, eye movements, and steering wheel operation, and alerts the driver to take a rest, thereby reducing the risk of accidents (58, 59).

##### 4.1.1.3 Comprehensive healthcare support

To enhance medical support, we planned to equip the vehicle with two key modules. The first was a daily health area featuring a locker stocked with various medicines, first aid kits, and stethoscopes. Commonly used medications could be stored in cabinets with compartmentalized drawers and clearly marked reminder labels. The second module was an emergency response system, which was designed to include SOS buttons at the bedside and in the bathroom; when activated, these buttons automatically send the user’s location to a designated contact person.

##### 4.1.1.4 Intelligent interactive interface

To ease operational challenges for older RV users, we planned to incorporate a high-definition touchscreen interface that supports intuitive gestures and tactile feedback. Additionally, an integrated high-precision voice recognition system could enable seamless voice command control. Considering the user’s concern for vehicle safety, we configured the interface to display real-time vehicle status, environmental monitoring data and navigation information to provide clear and instant vehicle driving information. We also designed the interface to be seamlessly connected with smart phones and cloud platforms to support remote monitoring, vehicle positioning and data synchronization.

##### 4.1.1.5 Durable materials

To avoid slipping under the feet of the older adults, we designed the vehicle flooring to be fully covered with non-slip PVC material. All furniture corners and door handles should be obtuse to prevent the older adults from being injured when bumping. The interior seats could be filled with high-density memory foam and covered with breathable and wear-resistant leather or stain-resistant fabrics, thus balancing comfort and durability. The exterior materials and coatings should be corrosion-resistant to ensure the beauty and durability of the vehicle’s appearance.

##### 4.1.1.6 Beautiful styling and well-coordinated colors

We designed the overall styling to be square and mature-looking, adopting a simple and smooth design language, avoiding overly complex surfaces, and presenting a clean and sophisticated visual style. Such a design aligns with older RV users’ pursuit of safety and practicality while improving space utilization. For color matching, we chose a calm and elegant black, complemented by other accent colors to create a harmonious and comfortable visual experience. The overall design would embody modernity and incorporate classic elements, making the vehicle better suited to the aesthetic preferences and daily needs of older RV users while maintaining its visual appeal.

#### 4.1.2 Refining sketch styling

Based on the key design elements identified through the Kano–AHP–QFD analysis, we further refined the functions and styling of the age-friendly RV.

First, the exterior styling should be developed in alignment with the quality attributes derived in the previous section. Based on these attributes, three preliminary design concepts were hand-sketched to explore potential styling directions. Then, for users to have a better concept visualization, renderings from the line drawings were created using an AI-powered website.^2^

Second, the interior design should follow the principles of comfort, safety, and age-friendliness, featuring four core functional zones: the sleeping area, living room, kitchen, and bathroom. In the sleeping area, a temperature regulation system was included to enhance sleep quality. In the bathroom, an optimized ventilation layout, dry-wet separation, and a non-slip textured floor were arranged to address both functional and safety needs. In the seating area, a skylight was placed to allow natural lighting and a rotatable table was configured to accommodate daily activities.

Third, for spatial planning, a modular layout and concealed storages can maximize the use of limited space. The passageway width was therefore laid out in compliance with China’s age-friendly standards (≥80 cm) (60) to minimize obstacles, complemented by curved furniture edges to reduce the risk of injury.

Apart from the above schemes, smart systems were integrated throughout the vehicle, including voice control, environmental monitoring and warning systems, and emergency call functions. One-touch alarm buttons and access to medical databases were planned in both the bathroom and living room, enabling quick symptom checks and remote consultations. Storage areas were organized according to the habits of older users, with a dedicated medicine freezer to support chronic disease management.

**Figure 7:**
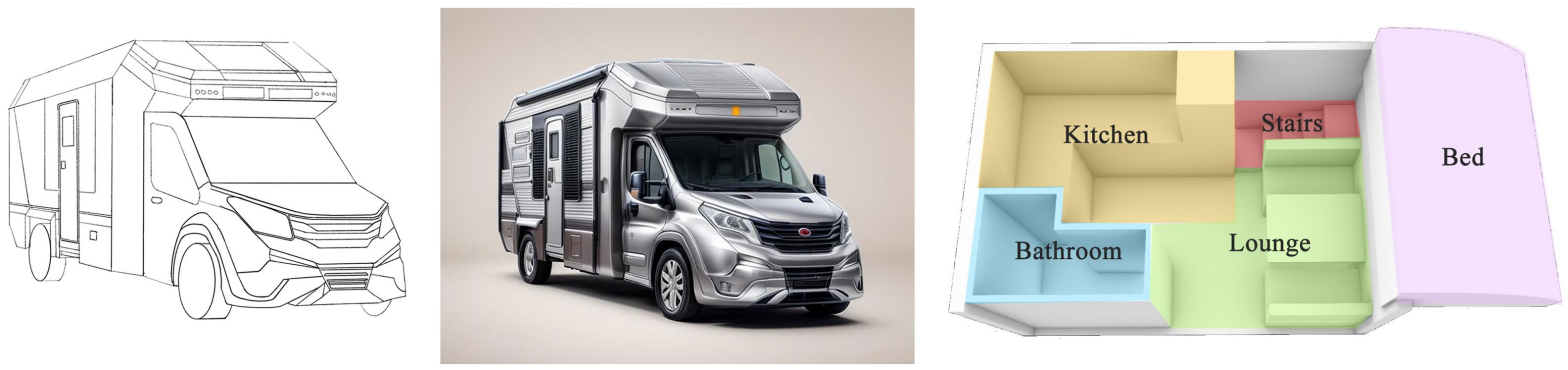
Sketch plan 1.

**Figure 8:**
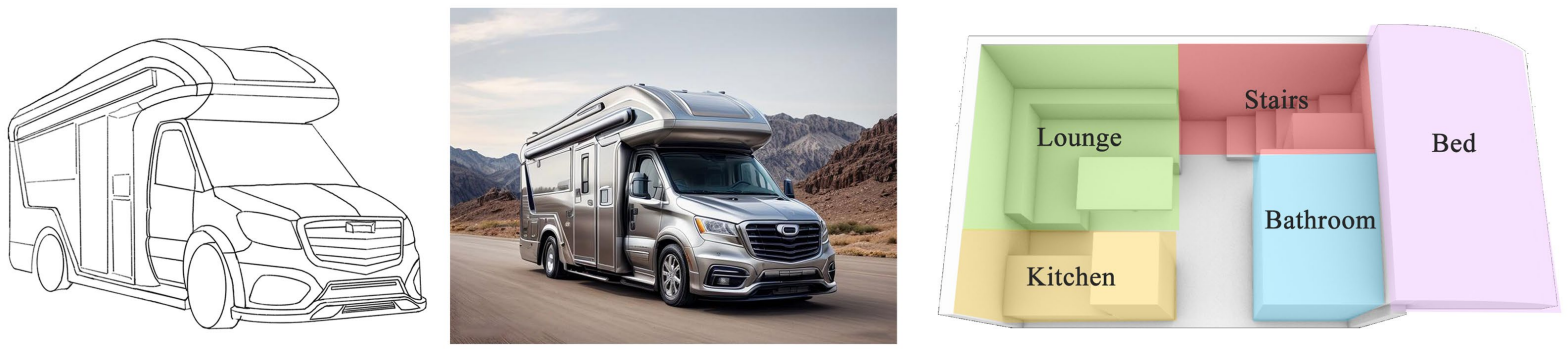
Sketch plan 2.

**Figure 9:**
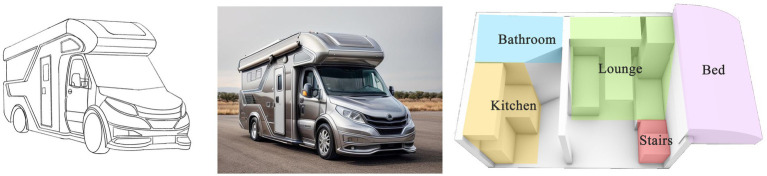
Sketch plan 3.

Accordingly, we developed three alternative interior layout schemes, as illustrated in Figures 7–9.

To support, rather than empirically validate, design decision-making by comparing how effectively each scheme met the identified core needs of older RV users (Figure 6), we used questionnaires to solicit opinions from 10 older RV users, 5 professors of industrial design, and 5 users with RV driving experience. Specifically, preferences based on the core user needs for living comfort, functional layout, intelligent control, age-friendly design, medical & safety assurance, and vehicle safety were measured using a five-point Likert scale: 1 – very dissatisfied. 2 – dissatisfied, 3 – unsure, 4 – satisfied, 5 – very satisfied. The descriptive statistics are shown in Table 8, allowing for a quick gathering of user feedback on the relative strengths and weaknesses of each design concept. This approach assumed normal distribution of responses and used mean scores to provide a clear, interpretable measure of user satisfaction.

**Table 8:** Evaluator satisfaction with the three age-friendly RV design schemes.

Scheme	Very satisfied	Satisfied	Unsure	Dissatisfied	Very dissatisfied	Mean
1	3 (15%)	4 (20%)	8 (40%)	1 (5%)	4 (20%)	3.05
2	0 (0%)	10 (50%)	6 (30%)	3 (15%)	1 (5%)	3.25
3	10 (50%)	5 (25%)	2 (10%)	2 (10%)	1 (5%)	4.05

Among the three design schemes, scheme 3 received the highest satisfaction rating (*M* = 4.05), with 75% of users selecting “satisfied” or “very satisfied.” In contrast, Scheme 1 had the lowest average score (*M* = 3.05) and the highest proportion of “unsure” (40%) and “very dissatisfied” (20%) responses, indicating weaker user acceptance. Scheme 2 ranked in between, with generally moderate evaluations. The highest satisfaction with Scheme 3 confirms that design schemes based on quantitative user demand analysis are more consistent with the real preferences of target users, avoiding the bias of “self-indulgent” design.

#### 4.1.3 Rendering

##### 4.1.3.1 Exterior rendering

**Figure 10:**
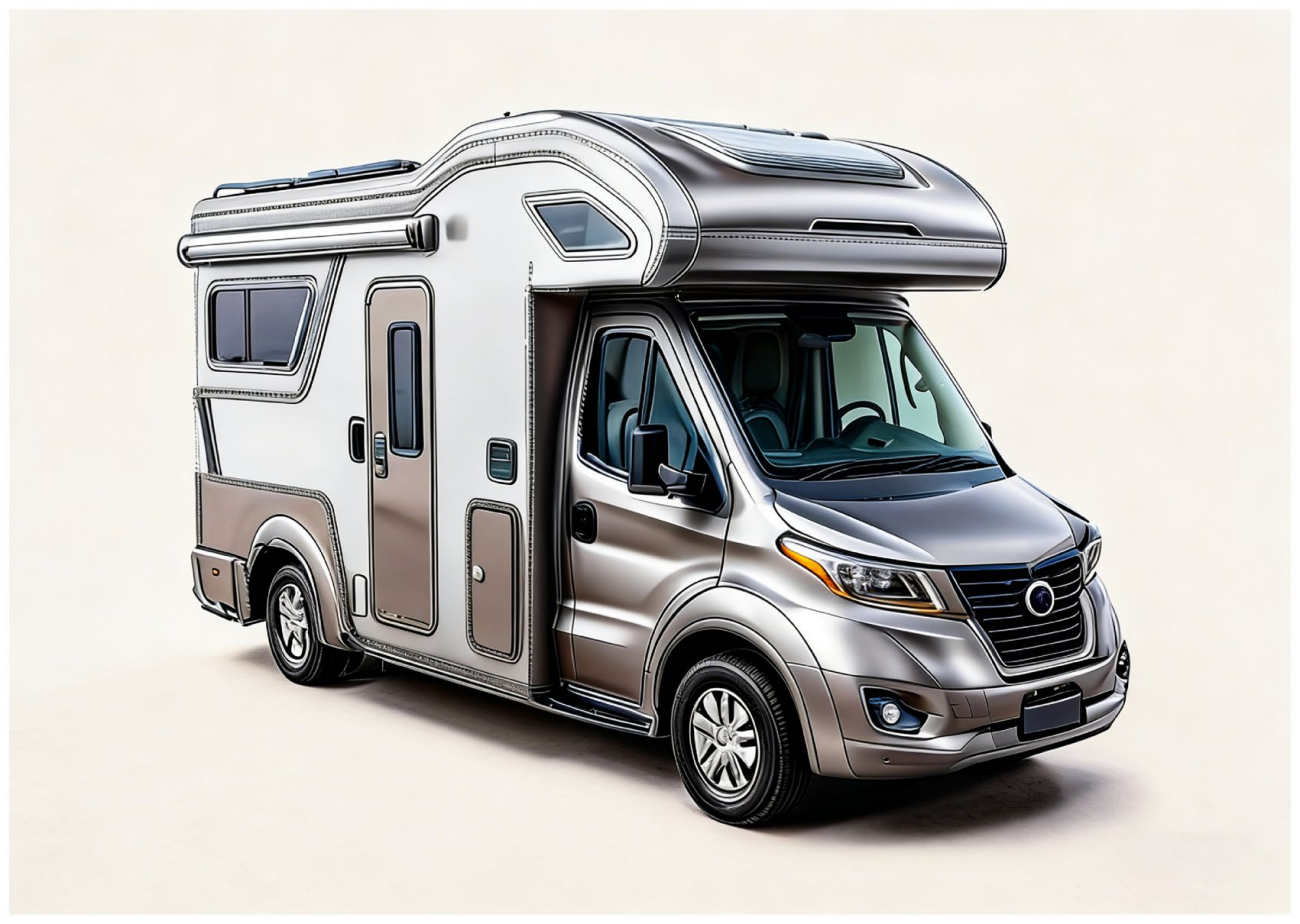
The rendering of the optimized design.

**Figure 11:**
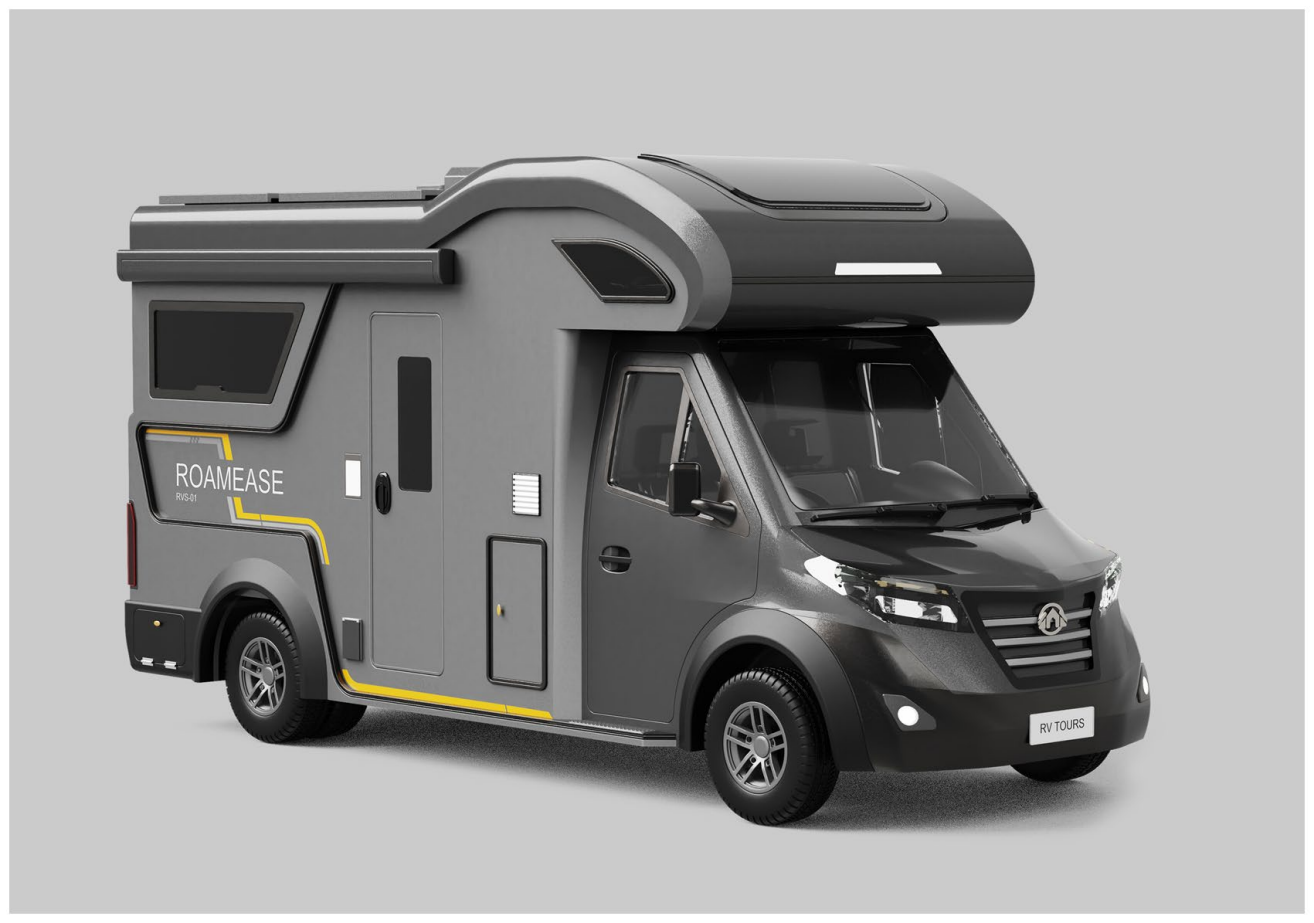
The rendering of the final scheme.

Based on the user evaluation results, scheme 3 was identified as the most satisfactory option, receiving the highest preference score among all three proposals. Building upon this scheme, we made further refinements through a systematic analysis of spatial layout, facility configuration, and styling proportions. The finalized exterior rendering is presented in Figure 10. Following that, we modeled the design scheme in 3D and then used KeyShot to apply to the model the materials, lighting, and scene rendering (Figure 11).

##### 4.1.3.2 Interior design

**Figure 12:**
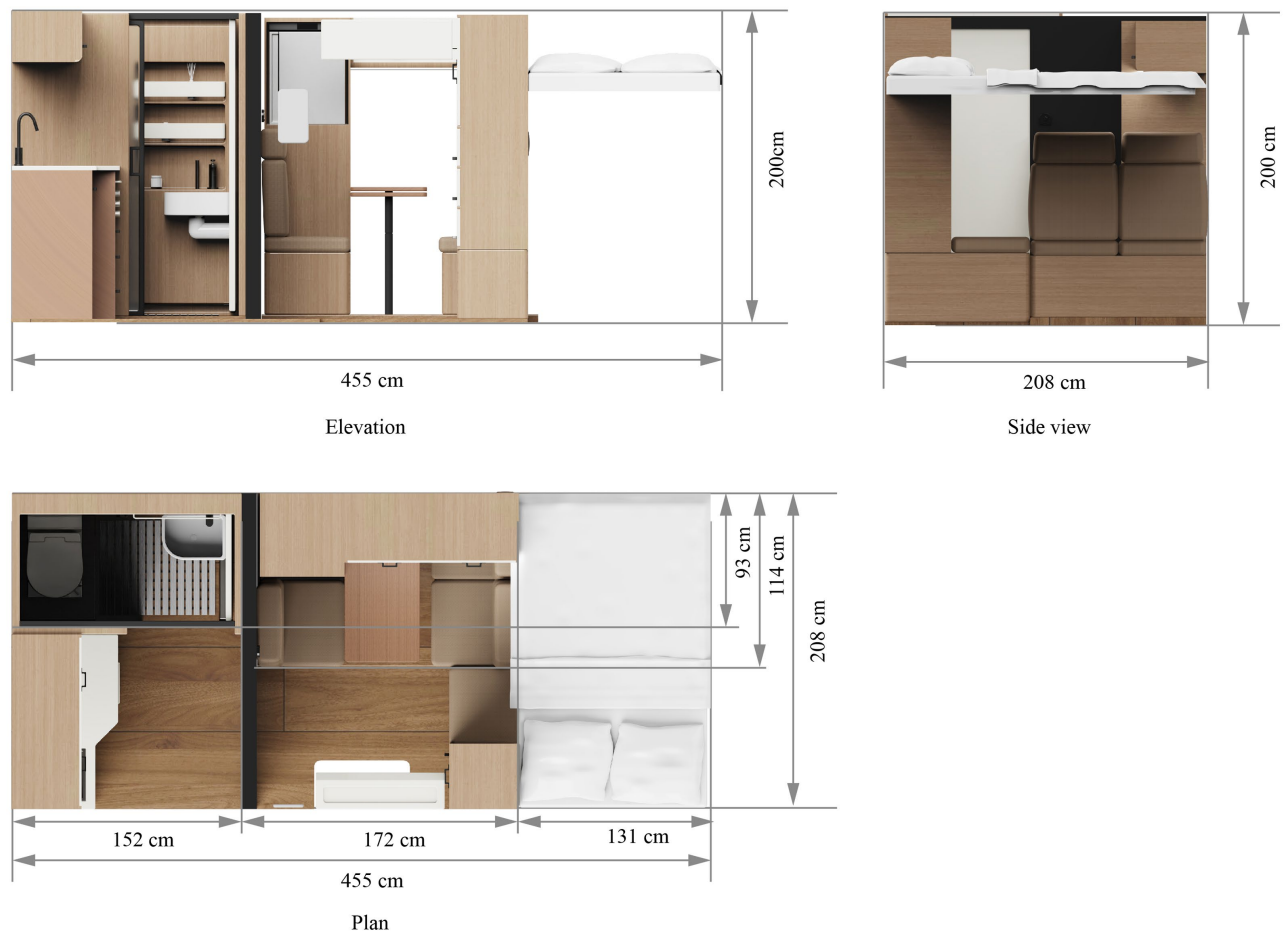
Size data.

Dimsensional data are illustrated in Figure 12. The RV has an interior length of 455 cm, a width of 208 cm, and a height of 200 cm. The bathroom measures 152 cm in length and 93 cm in width, while the kitchen measures 152 cm in length and 115 cm in width. The lounge area measures 172 cm in length and 208 cm in width, and the bed measures 208 cm in length and 131 cm in width.

**Figure 13:**
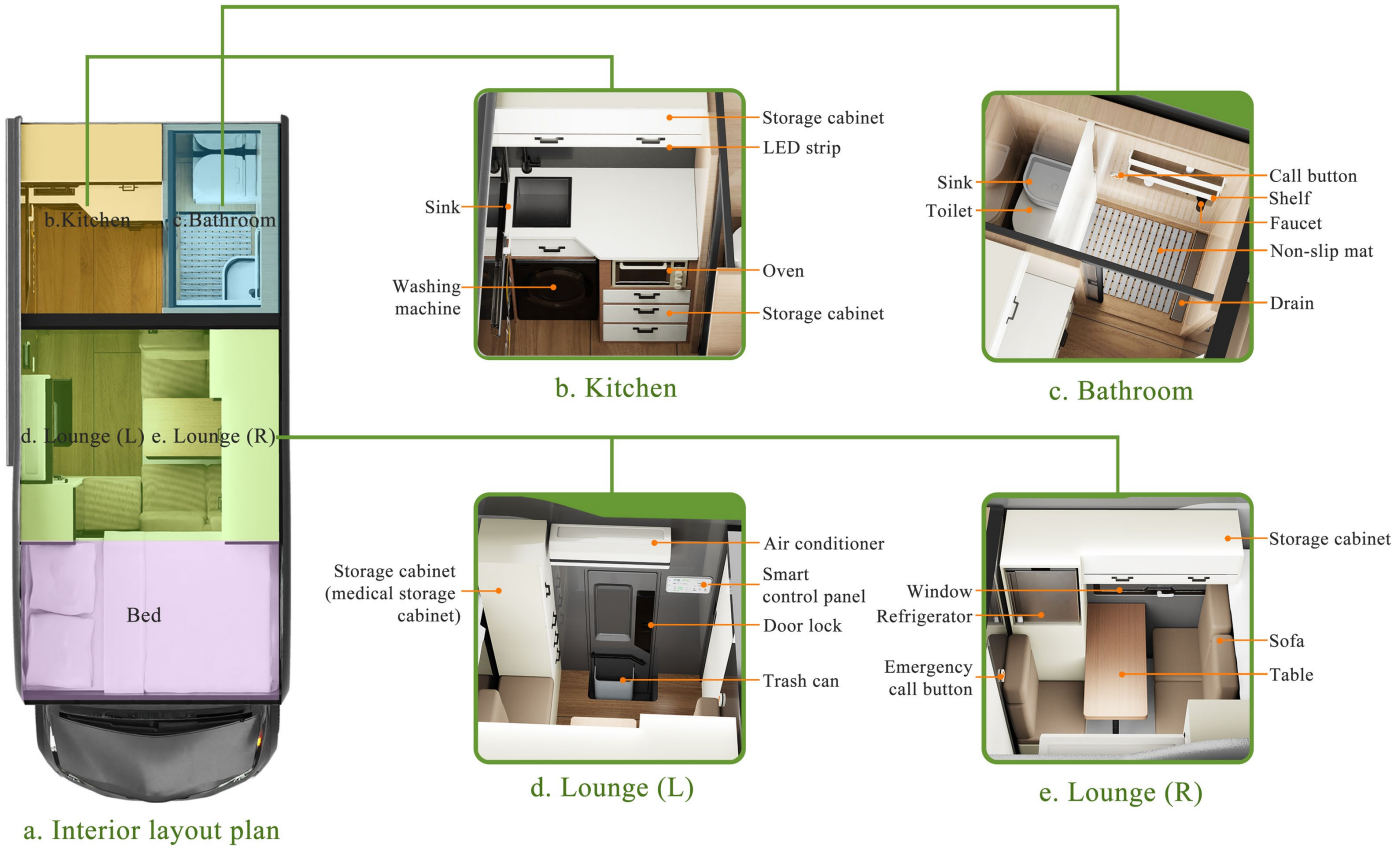
Functional zoning (**a**. Interior layout plan; **b**. Kitchen; c. Bathroom; **d**. Lounge (L); **e**. Lounge (R)).

Figure 13 further illustrates the layout and functions, with Figure 13a showing the interior layout plan, and the others providing details of each zone.

An independent kitchen (Figure 13b) was planned to isolate cooking fumes, with a side window for effective ventilation. Within the kitchen, tiered storage compartments above and base cabinets below were arranged to ensure both accessibility and spatial organization. Notably, in the cabinets, motion-sensing LED strips were added, providing automatic lighting in low-light environments to enhance cooking safety and efficiency. Moreover, a built-in oven was planned at mid-height for better visibility during baking, and space was reserved below for the installation of a washing machine.

For the bathroom (Figure 13c), we adopted a dry-wet separation layout, using a waterproof divider to physically separate the shower and toilet zones. Notably, for the bathroom flooring, anti-slip mats were planned, to prevent older RV users from slipping. Overhead, a bidirectional ventilation fan was designed to work in tandem with a concealed fresh air system, ensuring continuous air circulation and quick moisture removal. For added safety, a waterproof SOS emergency button was to be installed on the shower wall, which, when triggered, would activate the onboard alarm system and simultaneously send location data to a monitoring terminal. Overall, the structure aimed to strike a multidimensional balance among safety, hygiene, and comfort within a limited space.

On the right side of the lounge (Figure 13d), a dedicated medical compartment was allocated within a side cabinet for organizing chronic illness medication and first-aid tools. In addition, a wall-mounted smart control panel, supporting both touch and voice interaction, was designed to centrally manage air conditioning, lighting, and entertainment devices. Notably, the air conditioning system was planned to include a draft-free mode to avoid headaches in older RV users. Additionally, a hidden trash bin was incorporated into the door design to save space.

The left side of the lounge area (Figure 13e) was intended to feature a rotatable, foldable table that can be raised, lowered and extended to serve as a temporary bed. Also, a layered side cabinet was reserved to house a compact refrigerator, while panoramic sliding windows were planned on the adjacent wall, combining UV-resistant glass with built-in blackout curtains. Importantly, an emergency call button was to be placed behind the sofa, designed to trigger an audiovisual alarm and send location information to the monitoring terminal.

Overall, through spatial integration and multiple/redundant interactive mechanisms, our interior layout design supports a highly efficient and safe multi-dimensional living experience.

### 4.2 Designing the software interface for the age-friendly RV

**Figure 14:**
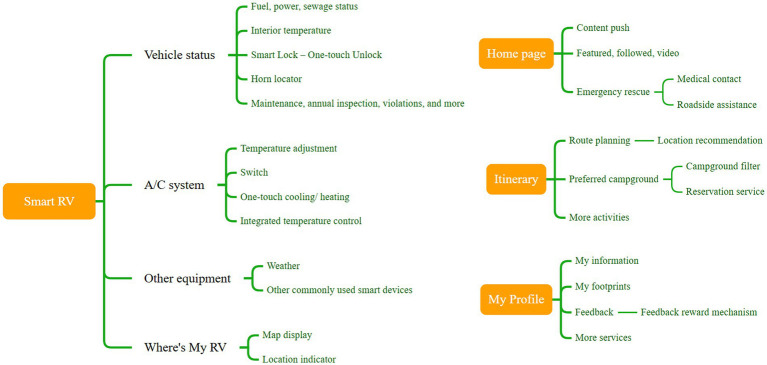
Information architecture of “RoamEase”.

Based on the key design points identified earlier, we outlined the corresponding functions, focusing primarily on RV features to establish the information architecture of the RoamEase app (Figure 14).

**Figure 15:**
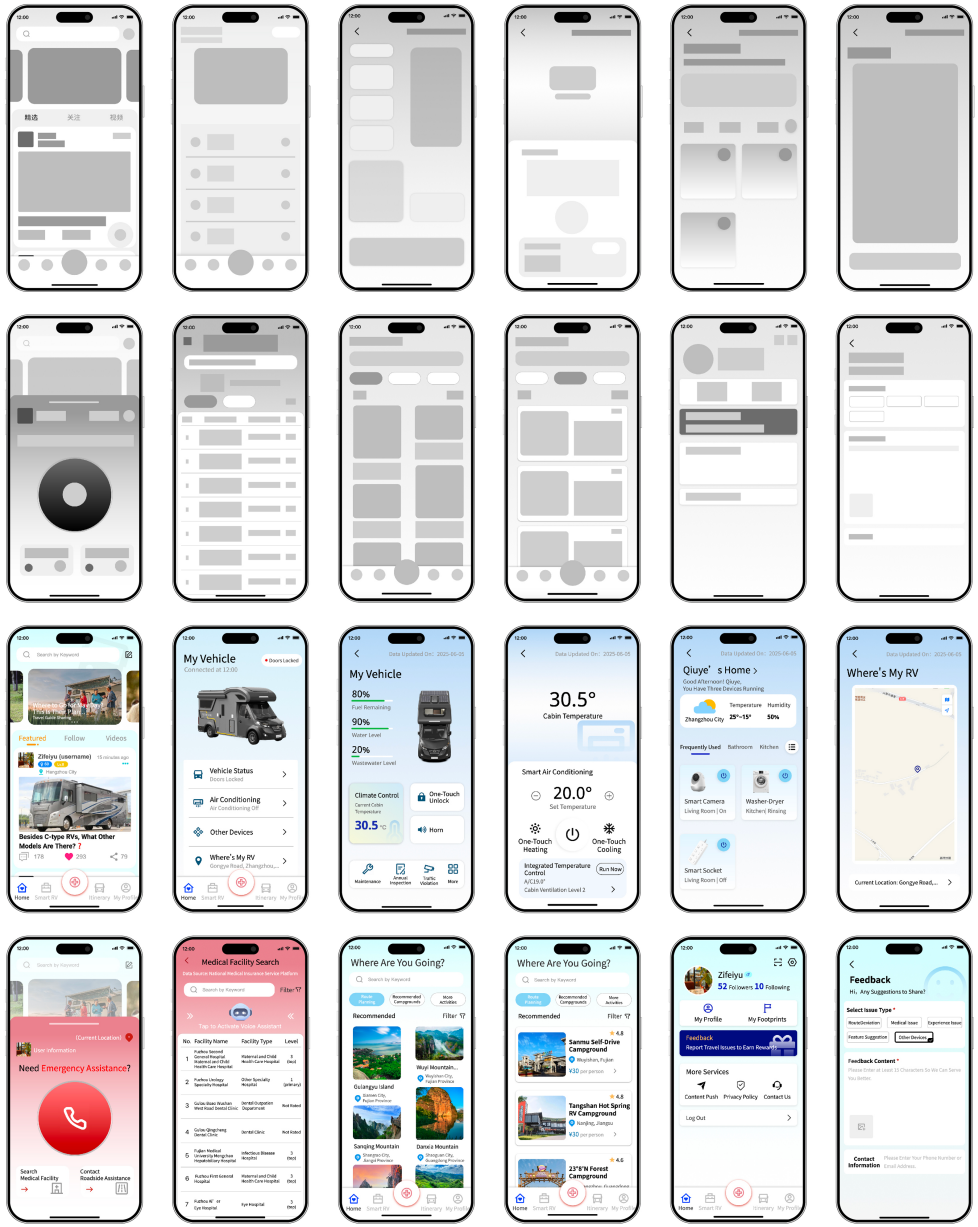
Examples of high-fidelity prototypes.

We began by developing low-fidelity prototypes, based on which we refined the details, replaced placeholder images with context-appropriate graphics, and adjusted some page layouts to create fully fleshed-out mockups. As for color matching, a calming blue palette—chosen to instill a sense of security—was accented with touches of orange to inject vitality. Throughout, we planned the interface to stay clean and uncluttered, ensuring that older users could quickly locate the buttons and features they need without being distracted by overly complex visuals. Several higher-fidelity prototypes of the mobile version are shown in Figure 15.

Specifically, in the “Home” page, users can browse and post information, fostering interaction among RV-based retirees and travel enthusiasts. An emergency-assistance feature was to be included in this interface, allowing one-tap calling of ambulance services, location-based search for nearby medical facilities, and direct contact with roadside rescue while prominently displaying the user’s profile and current GPS coordinates to expedite help in critical situations.

With the “Smart RV” page, users can monitor and control vehicle systems. For example, in the “Vehicle status” module, users can check fuel, fresh water, and wastewater levels, as well as operate the smart lock remotely to grant temporary access. In the air conditioning system, users could view the cabin temperature and choose between rapid-heat or rapid-cool modes; a combined temperature-control feature even allows temperature-sensitive users to set separate temperatures for the cockpit and living area.

In the “Other Devices” module, users can add and manage other smart appliances—such as security cameras, the washing machine, or smart outlets—directly from the app. The “Where’s My RV” feature helps users to prevent lost-vehicle incidents by showing the RV’s position. The “Itinerary” page presents system-recommended attractions with filtering options, and once a destination is selected, the app generates both driving directions and a tailored travel plan. The “Preferred Campgrounds” search function provides information on nearby rest and service areas, making it easy to plan stops for vehicle maintenance or supplies. Finally, in the “My Profile” page, users can edit their personal information and access additional app services.

**Figure 16:**
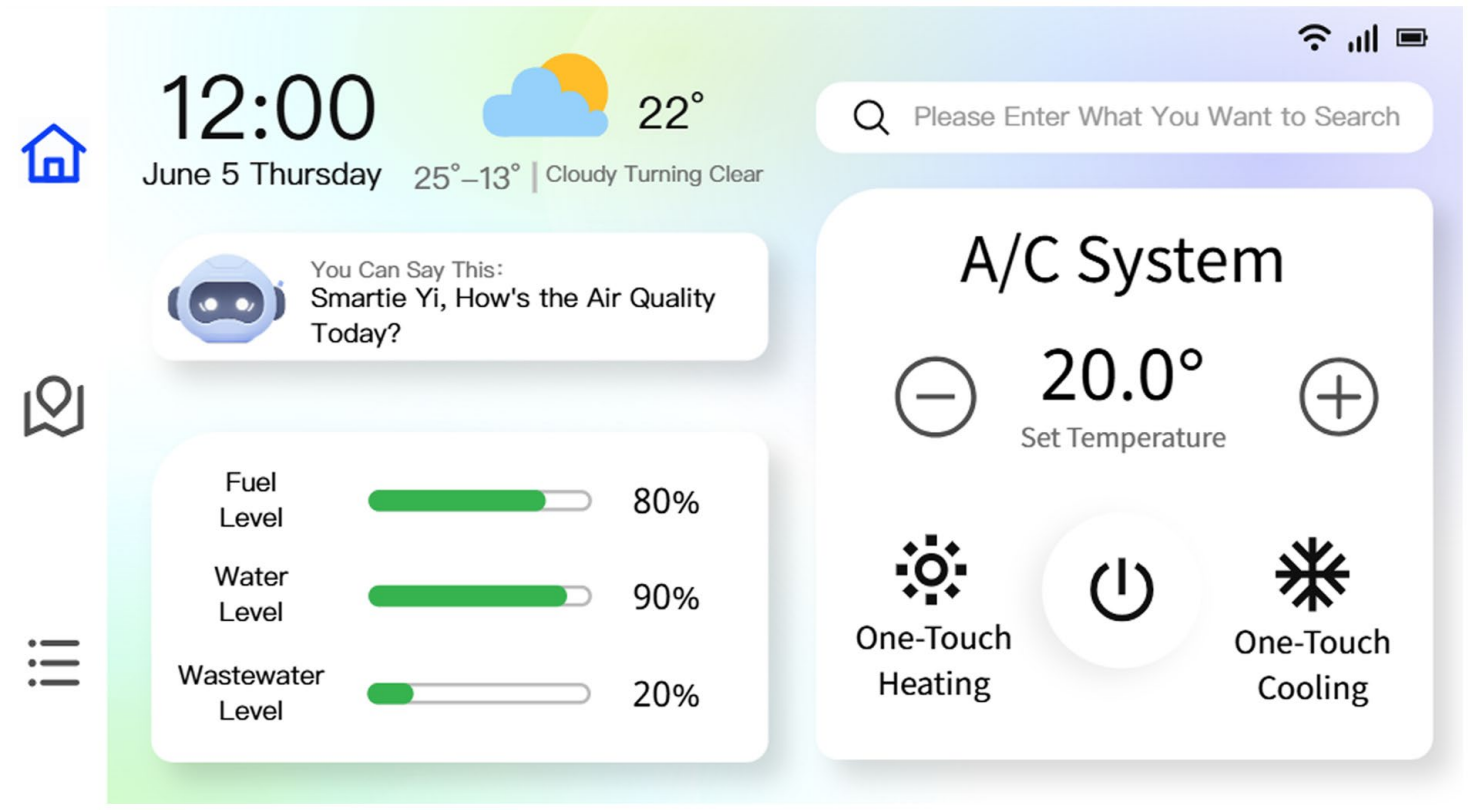
A selected display of the interior interactive interface.

In addition to the mobile app, we also designed a multimodal interactive control terminal (touch + voice), which integrates core system monitoring and equipment management functions. It displays real-time data on fuel, fresh water, and wastewater levels, and supports multi-mode air conditioning control, including automatic activation or shutdown based on preset temperature thresholds. Users can activate a voice assistant to issue commands verbally. The system can also load high-precision offline maps, marking older-adult-friendly service facilities such as medical stations and accessible restrooms, as shown in Figure 16.

## 5 Discussions and conclusion

The pressing reality of China’s “growing old before getting rich” has been driving the country toward more diversified older adult care models, one of which is RV-based retirement. There is, however, scarce research on whole-vehicle design of age-friendly RVs. This study addresses this supply–demand mismatch by developing an integrated Kano–AHP–QFD analytical framework to systematically optimize the entire process from user needs identification to product function transformation.

Specifically, through in-depth interviews combined with Kano model analysis, we systematically identified, for the first time, 34 core needs across five dimensions of age-friendly RV design. This reveals that neglecting 8 must-be (*M*) needs would sharply reduce user satisfaction, whereas fulfilling 9 attractive (*A*) needs would greatly enhance it. Building on this, we employed the AHP to quantify and prioritize key needs, establishing a clear hierarchy: *comfortable sleep > vehicle operation > bathroom ventilation*. Subsequently, by applying QFD and the House of Quality, we translated these user needs into technical priorities. This resulted in a ranked list of ten key technical characteristics, with *living comfort > age-friendly design > vehicle safety assurance* at the top. Overall, our design process formed a chain-like transformation mechanism of *needs stratification – priority quantification – function mapping*.

Guided by this methodological framework, we proposed an innovative RV design scheme focusing on barrier-free spatial accessibility, intelligent driver assistance, and integrated medical support. Key features, such as passageways at least 80 cm wide for wheelchair access and a dry–wet separated bathroom with anti-slip flooring, would enhance safety and ensure the quality of travel-based retirement living. These features are meaningful to promoting travel-based older adults care, as safety is a major concern for older people on travel (20), long-distance travel in particular. They also provide implications for the limited studies on older-adult-focused RV campsite optimization (61), for example, to mandate age-friendly facilities and maximize attractiveness (62), based on Kano-modeled “must-be” needs such as barrier-free access (e.g., corridors at least corridors at least 80/90/91.5 cm wide according to China’s (60), UN’s (63) and USA’s (64) guidelines).

In addition, a dual-module healthcare system that links bedside SOS buttons with real-time health monitoring (65) not only meets older adults’ practical healthcare needs but also provides them with psychological reassurance, addressing both daily care and emergency concerns. Moreover, the ADAS, complemented by the “RoamEase” RV mobile app and onboard interaction system, extends intelligent control and safety assurance functions, thus addressing ADAS acceptance through intuitive and senior-friendly user interfaces (54) and enriching the overall travel experience for older adults.

Overall, our findings align with Li and Li (44) emphasizing the importance of age-friendly design in medical devices. However, our study extends this perspective to mobile living environments, revealing that older RV users prioritize “must-be” needs such as comfortable sleeping spaces (*n*_1_) and vehicle operation simplicity (*n*_33_) due to physiological changes associated with aging, including reduced mobility and cognitive function. This contrasts with Marques & Rodrigues (12) who found that European motorhome travelers prioritize social interaction, highlighting cultural differences in user needs. Unlike Wu (23) who focused on post-design user feedback, our Kano–AHP–QFD framework proactively identifies unmet needs before design implementation. Psychologically, this approach addresses the “fear of the new” often experienced by older RV users (20) by prioritizing familiar, intuitive features such as large touch-screen interfaces and voice control.

Methodologically, unlike most existing RV research, which primarily employs methods like AHP (27), FAHP-FBS integration (28), and affordance theory (25), to design partial components such as interior layout (24) and styling (27), our closed-loop Kano-AHP-QFD approach hierarchically classifies older adults needs (Kano), quantifies priorities (AHP), and maps to holistic design features (QFD). Rather than functioning as independent modules, these stages operate as a sequentially constrained system in which the output of each stage structurally shapes the next. Our framework reveals that weighting allocation and needs–function mapping constitute critical leverage mechanisms within multi-stage decision integration. While all stages are interdependent, variations in priority weighting and relationship intensity directly influence downstream design rankings, thereby shaping final design outcomes. This suggests that in complex product-system design, the cumulative integration of filtering, weighting, and translation mechanisms may be more consequential than the isolated use of any single analytical tool.

Regarding contributions, our research extends Liu et al.’s (45) Kano–AHP–QFD-based scope from single-interface optimization to a holistic living-mobile ecosystem integrating exterior styling, interior layout, and interactive systems, thus contributing to the “healthcare-on-wheels” lifestyle. Additionally, our research may provide insights for (1) older-adult-oriented retrofit or renovation of RVs (e.g., non-slip PVC flooring; curved protective covers for furniture corners and door handles; large touch-screen control panel replacing traditional knobs) and (2) a cross-sector collaboration platform linking automotive enterprises, healthcare, and cultural tourism, to ensure a carefree travel-based retirement lifestyle.

Despite its merits, this research has several limitations. First, a key limitation is the small sample size (15 older adults participants) due to the proximity-based recruitment. Though pragmatic, this sampled group may introduce geographical clustering bias, as participants were locally concentrated despite diverse origins. Future research could expand sampling across multiple regions and integrate mixed-methods (e.g., cross-regional surveys) to enhance the generalizability of age-friendly RV user needs. Second, the QFD analysis relies on expert ratings to determine the strength of relationships between user needs and technical characteristics. While this is a common practice, the results are inherently subjective and reflect the collective expertise and perspectives of our expert panel. This subjectivity means the QFD outcomes should be interpreted as a guide rather than a definitive scheme. Future research could thus explore alternative methods such as machine learning-based correlation analysis to enhance objectivity. Third, the comparison of the three design schemes was based on a relatively small sample and relied primarily on descriptive statistics. As such, the analysis functioned as a decision-support exercise rather than a rigorous empirical validation. The findings should therefore be interpreted within the scope of the core user needs identified through the Kano–AHP–QFD framework. Future research could use larger sample sizes and employ inferential statistics such as ANOVA and Chi-square test to provide more robust empirical validation of the design schemes. Fourth, the current health support module remains basic, lacking integration with advanced AI and embodied intelligence for proactive health management. Future research may integrate “large models + embodied intelligence” to develop AI health management assistants and introduce unobtrusive monitoring technologies to enable dynamically adaptive, proactive iterations.

## Data Availability

The original contributions presented in the study are included in the article/supplementary material, further inquiries can be directed to the corresponding author.

## Footnotes

1
https://turboscribe.ai/

2
https://www.bigbigai.com/

## References

[1] Navaneetham K Arunachalam D. "Global population aging, 1950–2050". In: Handbook of Aging, Health and Public Policy. Singapore: Springer (2023)

[2] Lobanov-Rostovsky S He Q Chen Y Liu Y Wu Y Liu Y . Growing old in China in socioeconomic and epidemiological context: systematic review of social care policy for older people. BMC Public Health. (2023) 23:1272. doi: 10.1186/s12889-023-15583-1, 37391766 PMC10311713

[3] Luo Y Su B Zheng X. Trends and challenges for population and health during population aging—China, 2015–2050. China CDC Weekly. (2021) 3:593–8. doi: 10.46234/ccdcw2021.158, 34594944 PMC8393078

[4] Xu Y Hu T Wang J. How do older parents with one child live? The well-being of Chinese elders affected by the one-child policy. SAGE Open. (2024) 14. doi: 10.1177/21582440241253406

[5] Chen H Hagedorn A An N. The development of smart eldercare in China. Lancet Regional Health. (2023) 35:100547. doi: 10.1016/j.lanwpc.2022.100547, 37424692 PMC10326707

[6] Cole S Hua C Peng S Wang W. Exploring the relationship of leisure travel with loneliness, depression, and cognitive function in older adults. Int J Environ Res Public Health. (2024) 21:498. doi: 10.3390/ijerph21040498, 38673409 PMC11050658

[7] Tang J Yan L Wang Y Huang L. Psychological restoration and transformative experiences of recreational vehicle tourists. Curr Issues Tour. (2024) 29:338–53. doi: 10.1080/13683500.2024.2420849

[8] Hu F Wen J Phau I Ying T Aston J Wang W. The role of tourism in healthy aging: an interdisciplinary literature review and conceptual model. J Hosp Tour Manag. (2023) 56:356–66. doi: 10.1016/j.jhtm.2023.07.013

[9] Gu D Zhu H Brown T Hoenig H Zeng Y. Tourism experiences and self-rated health among older adults in China. J Aging Health. (2016) 28:675–703. doi: 10.1177/0898264315609906, 26486781 PMC5381654

[10] Li F Wang D Liu C Sun F. Spatial distribution characteristics and mechanistic drivers of self-driving and RV camping in China. Resour Sci. (2017) 39:288–302. doi: 10.18402/resci.2017.02.11

[11] Fox MA Lane LD Black G. Competitive forces in the United States recreational vehicle industry. SSRN Electron J. (2011). doi: 10.2139/ssrn.1578225

[12] Marques JF Rodrigues TI. Enjoyers’, ‘seekers’ and ‘vacationers’: proposal for a typology of motorhome travellers in Europe. Tourist Stud. (2024) 24:172–94. doi: 10.1177/14687976241228008

[13] Jinan Daily. (2024). Why seniors are hitting the road in RVs at 80? Jinan Daily. Available online at: https://baijiahao.baidu.com/s?id=1808667827635291890&wfr=spider&for=pc (Accessed January 2, 2025)

[14] Wang E de Bono A Wong I. A case study: designing a sustainable recreational vehicle for the emerging market through computer-aided design process. Comput Aided Des Appl. (2014) 11:S27–35. doi: 10.1080/16864360.2014.914404

[15] Yu R. (2023). Recreational vehicles take to the roads in numbers: Ownership rises as more families seek diverse lifestyles. China Daily, 23 February. Available online at: http://www.chinadaily.com.cn/a/202302/23/WS63f6d34da31057c47ebb0707.html (Accessed January 7, 2025)

[16] Zhang X Wang X Xu W. Research on user demands and functional design of an AR-based interior design and display platform for recreational vehicles. Appl Sci. (2024) 14:10568. doi: 10.3390/app142210568

[17] Gong J Guo X Qi C Pan L Liu X. Research on assessing driving ability of older drivers based on cognitive tests: a case study of Beijing, China. Sustainability. (2023) 15:3031. doi: 10.3390/su15043031

[18] Tretter RE Nies MA Omotowa OO. Recreational-vehicle-dwelling American nomads’ experiences seeking healthcare: a qualitative field study. J Adv Nurs. (2024) 80:2893–904. doi: 10.1111/jan.16033, 38131510

[19] Bugg JM Zook N Delosh EL Davalos DB Davis HP. Age differences in fluid intelligence: contributions of general slowing and frontal decline. Brain Cogn. (2006) 62:9–16. doi: 10.1016/j.bandc.2006.02.006, 16603300

[20] Kim S Sivangula P. Toward safe and confident silver drivers: interview study investigating older adults’ driving practices. JMIR Aging. (2024) 7:e57402. doi: 10.2196/57402, 39133531 PMC11347888

[21] Xia Y Cao M. RV design strategies for new old men driven by tourism experience. Design. (2024) 37:140–3. doi: 10.20055/j.cnki.1003-0069.001585

[22] Li T. Research on interaction design of shared RV based on user needs. Toys World. (2024) 2:189–92.

[23] Wu M. Comfort, convenience, and development trends in RV design. Mach China. (2023) 2023:85–8.

[24] Chen G Liu Y Shen Z Zhang F. Ergonomic design of kitchen space of self-propelled C-type recreational vehicles. Packag Eng. (2021) 42:119–125, 154. doi: 10.19554/j.cnki.1001-3563.2021.14.013

[25] Lin X Xu J Ren Z. Research on interior space design of RV based on affordance theory. Design. (2023) 36:63–5.

[26] Niu Q Cheng S Qiu Z. Design method of small recreational vehicle’s interior space based on user behavior data analysis. Symmetry. (2025) 17:2096. doi: 10.3390/sym17122096

[27] Xu X Cheng Y Chen G. Evaluation method and application of RV modeling based on AHP method. J Mach Des. (2020) 37:140–4. doi: 10.13841/j.cnki.jxsj.2020.06.022

[28] Tu B Li X. Research on self-contained RV onboard human–machine interface design based on FAHP-FBS integration. Design. (2024) 10:139–41. doi: 10.16272/j.cnki.cn11-1392/j.2024.10.014

[29] Kano N Seraku N Takahashi F Tsuji S. Attractive quality and must-be quality. J Japan Soc Qual Control. (1984) 14:147–56. doi: 10.20684/quality.14.2_147

[30] Hartono M Chuan TK. How the Kano model contributes to Kansei engineering in services. Ergonomics. (2011) 54:987–1004. doi: 10.1080/00140139.2011.616229, 22026943

[31] Wu M Wang L. A continuous fuzzy Kano’s model for customer needs analysis in product development. Proc Inst Mech Eng B J Eng Manuf. (2011) 226:535–46. doi: 10.1177/0954405411414998

[32] Slevitch L. Kano model categorization methods: typology and systematic critical overview for hospitality and tourism academics and practitioners. J Hospital Tourism Res. (2024) 49:449–79. doi: 10.1177/10963480241230957

[33] Neira-Rodado D Ortíz-Barrios M la De Hoz-Escorcia S Paggetti C Noffrini L Fratea N. Smart product design process through the implementation of a fuzzy Kano-AHP-DEMATEL-QFD approach. Appl Sci. (2020) 10:1792. doi: 10.3390/app10051792

[34] Jin J Jia D Chen K. Mining online reviews with a Kansei-integrated Kano model for innovative product design. Int J Prod Res. (2021) 60:6708–27. doi: 10.1080/00207543.2021.1949641

[35] Li J Shen Z Chen Y Liu Y Lin Z Fei N. Research on the age-friendly design of station square based on IPA-Kano model: a case study of Chengdu east Railway Station. J Asian Architect Build Eng. (2025) 25:1538–53. doi: 10.1080/13467581.2025.2472712

[36] Lin J Li X Lin J. Evaluation of age-appropriate public seats in comprehensive parks and sustainable design strategies based on the Kano–importance–performance analysis model. Sustainability. (2024) 16:6914. doi: 10.3390/su16166914

[37] Tu E Zhou Y. A grounded theory based Kano model to develop age-friendly housing retrofit strategies for older people in China. J Asian Architect Build Eng. (2025) 25:–304. doi: 10.1080/13467581.2025.2455037

[38] Wang T Zhao Y Zhang X Xie Y Pang LLL. Design of walking aids for the elderly based on the Kano-AHP-FEC method. Sci Rep. (2025) 15:2663. doi: 10.1038/s41598-025-85540-y, 39837980 PMC11751321

[39] Jing Y Cheng Y Zhu L Xu X. User needs-driven research and design validation of electric vehicles for the elderly. Packag Eng. (2024) 45:56–64. doi: 10.19554/j.cnki.1001-3563.2024.22.006

[40] Saaty TL. The Analytic Hierarchy Process: Planning, Priority Setting, Resource Allocation. New York: McGraw-Hill (1980).

[41] Saaty TL. Decision making with the analytic hierarchy process. Int J Serv Sci. (2008) 1:83–98. doi: 10.1504/ijssci.2008.017590

[42] Emrouznejad A Marra M. The state of the art development of AHP (1979–2017): a literature review with a social network analysis. Int J Prod Res. (2017) 55:6653–75. doi: 10.1080/00207543.2017.1334976

[43] Vaidya OS Kumar S. Analytic hierarchy process: an overview of applications. Eur J Oper Res. (2006) 169:1–29. doi: 10.1016/j.ejor.2004.04.028

[44] Li X Li H. Age-appropriate design of domestic intelligent medical products: An example of smart blood glucose detector for the elderly with AHP-QFD joint KE. Heliyon. (2024) 10:e27387. doi: 10.1016/j.heliyon.2024.e27387, 38486754 PMC10937709

[45] Liu W Li Y Cai J. Aging design of passenger car center control interface based on Kano/AHP/QFD models. Electronics. (2024) 13:5004. doi: 10.3390/electronics13245004

[46] Mizuno S Akao Y. QFD: The Customer-driven Approach to Quality Planning and Deployment. Tokyo: Asian Productivity Organization (1994).

[47] Rianmora S Sukjitsakunchai L Tachawatanawisal T. Designing and developing a platform called ‘rest at restaurant’ to support the basic needs of senior adults. Cogent Bus Manag. (2024) 11. doi: 10.1080/23311975.2024.2301798

[48] Violante MG Vezzetti E. Kano qualitative vs quantitative approaches: an assessment framework for products attributes analysis. Comput Ind. (2017) 86:15–25. doi: 10.1016/j.compind.2016.12.007

[49] Anthony K Miller-Day M Dupuy M Ventura J Hodges AL Alonso-Pecora D . Is there really a difference? A comparison of in-person and online qualitative interviews. Int J Qual Methods. (2025) 24. doi: 10.1177/16094069251349580

[50] Paulhus DL. "Measurement and control of response bias". In: Robinson JP Shaver PR Wrightsman LS, editors. Measures of Personality and Social Psychological Attitudes. San Diego: Academic Press (1991). p. 17–59.

[51] Matzler K Hinterhuber HH Bailom F Sauerwein E. How to delight your customers. J Prod Brand Manag. (1996) 5:6–18. doi: 10.1108/10610429610119469

[52] Saaty TL Vargas LG. Decision Making with the Analytic Network Process. New York: Springer (2006).

[53] Yang C Cheng J Wang X. Hybrid quality function deployment method for innovative new product design based on the theory of inventive problem solving and Kansei evaluation. Adv Mech Eng. (2019) 11. doi: 10.1177/1687814019848939

[54] Liang D Lau N Baker SA Antin JF. Examining senior drivers’ attitudes toward advanced driver assistance systems after naturalistic exposure. Innov Aging. (2020) 4:igaa017. doi: 10.1093/geroni/igaa017, 32582868 PMC7302428

[55] Xiao L Gao F. A comprehensive review of the development of adaptive cruise control systems. Veh Syst Dyn. (2010) 48:1167–92. doi: 10.1080/00423110903365910

[56] Awaisi KS Abbas A Khattak HA Ahmad A Ali M Khalid A. Deep reinforcement learning approach towards a smart parking architecture. Clust Comput. (2023) 26:255–66. doi: 10.1007/s10586-022-03599-y

[57] Yang J Coughlin JF. In-vehicle technology for self-driving cars: advantages and challenges for aging drivers. Int J Automot Technol. (2014) 15:333–40. doi: 10.1007/s12239-014-0034-6

[58] Jia H Xiao Z Ji P. Fatigue driving detection based on deep learning and multi-index fusion. IEEE Access. (2021) 9:147054–62. doi: 10.1109/ACCESS.2021.3123388

[59] Tian Y Cao J. Fatigue driving detection based on electrooculography: a review. Eurasip J Image Video Process. (2021) 2021:33. doi: 10.1186/s13640-021-00575-1

[60] Ministry of Housing and Urban-Rural Development of the People’s Republic of China. (2012). Codes for accessibility design. Available online at: https://www.chinesestandard.net/PDF.aspx/GB50763-2012 (Accessed January 2, 2025)

[61] Dai X Zhang Q. Optimizing spatial layout of campsites for self-driving tours in Xinjiang: a study based on online travel blog data. Sustainability. (2024) 16:4176. doi: 10.3390/su16104176

[62] Thiel D Leroux E Labarbe E. Optimizing shared recreational vehicle service areas: a multi-strategy approach for economic performance and user satisfaction. Tour Econ. (2023) 30:1465–91. doi: 10.1177/13548166231214573

[63] United Nations. (2003). Accessibility for the disabled: A design manual for a barrier-free environment. Available online at: https://www.un.org/esa/socdev/enable/designm/AD2-09.htm (Accessed January 2, 2025)

[64] U.S. Department of Justice Civil Rights Division. (1991). ADA Standards for Accessible design: Title III Regulation 28 CFR Part 36. Washington, DC: U.S. Department of Justice. Available online at: https://www.ada.gov/law-and-regs/design-standards/1991-design-standards/ (Accessed January 2, 2025)

[65] Abdulmalek S Nasir A Jabbar WA Almuhaya MAM Bairagi AK Khan MA-M . IoT-based healthcare-monitoring system towards improving quality of life: a review. Healthcare. (2022) 10:1993. doi: 10.3390/healthcare10101993, 36292441 PMC9601552

[66] Ficalora JP Cohen L. Quality Function Deployment and Six Sigma: A QFD Handbook. 2nd ed. Upper Saddle River, NJ: Prentice Hall. (2009).

